# Biomass, lipid accumulation kinetics, and the transcriptome of heterotrophic oleaginous microalga *Tetradesmus bernardii* under different carbon and nitrogen sources

**DOI:** 10.1186/s13068-020-01868-9

**Published:** 2021-01-06

**Authors:** Baoyan Gao, Feifei Wang, Luodong Huang, Hui Liu, Yuming Zhong, Chengwu Zhang

**Affiliations:** 1 Department of Ecology, Research Center for Hydrobiology, Jinan University, Guangzhou, 510632 People’s Republic of China; 2 College of Resources and Environment, Zhongkai University of Agriculture and Engineering, Guangzhou, 510225 China

**Keywords:** *Tetradesmus bernardii*, Heterotrophy, Oleaginous microalgae, C/N ratio, Transcriptome

## Abstract

**Background:**

Heterotrophic cultivation of microalgae has been proposed as a viable alternative method for novel high-value biomolecules, enriched biomass, and biofuel production because of their allowance of high cell density levels, as well as simple production technology. *Tetradesmus bernardii*, a newly isolated high-yielding oleaginous microalga under photoautotrophic conditions, is able to grow heterotrophically, meaning that it can consume organic carbon sources in dark condition. We investigated the effect of different carbon/nitrogen (C/N) ratios on the growth and lipid accumulation of *T. bernardii* in heterotrophic batch culture under two nitrogen sources (NaNO_3_ and CO(NH_2_)_2_). In addition, we conducted time-resolved transcriptome analysis to reveal the metabolic mechanism of *T. bernardii* in heterotrophic culture.

**Results:**

*T. bernardii* can accumulate high biomass concentrations in heterotrophic batch culture where the highest biomass of 46.09 g/L was achieved at 100 g/L glucose concentration. The rate of glucose to biomass exceeded 55% when the glucose concentration was less than 80 g/L, and the C/N ratio was 44 at urea treatment. The culture was beneficial to lipid accumulation at a C/N ratio between 110 and 130. NaNO_3_ used as a nitrogen source enhanced the lipid content more than urea, and the highest lipid content was 45% of dry weight. We performed RNA-seq to analyze the time-resolved transcriptome of *T. bernardii*. As the nitrogen was consumed in the medium, nitrogen metabolism-related genes were significantly up-regulated to speed up the N metabolic cycle. As chloroplasts were destroyed in the dark, the metabolism of cells was transferred from chloroplasts to cytoplasm. However, storage of carbohydrate in chloroplast remained active, mainly the synthesis of starch, and the precursor of starch synthesis in heterotrophic culture may largely come from the absorption of organic carbon source (glucose). With regard to lipid metabolism, the related genes of fatty acid synthesis in low nitrogen concentration increased gradually with the extension of cultivation time.

**Conclusion:**

*T. bernardii* exhibited rapid growth and high lipid accumulation in heterotrophic culture. It may be a potential candidate for biomass and biofuel production. Transcriptome analysis showed that multilevel regulation ensured the conversion from carbon to the synthesis of carbohydrate and lipid.

## Introduction

Microalgae are generally photoautotrophic organisms, but several can grow heterotrophically, exhibiting considerable metabolic flexibility and versatility [1]. Heterotrophically growing microalgae absorb exogenous organic carbon as the source of energy instead of light. Compared to photoautotrophy, heterotrophic cultivation can significantly increase growth rate and biomass productivity, eliminate the light dependence, and be easier to control monoculture and scale up [2]. Due to these advantages, hetertrophic production of valuable products, including pigments, fatty acids, pharmaceuticals, and biofuels, has received substantially increasing interest [3–5]. Under heterotrophic culture, many microalgae, such as *Haematococcus pluvialis*, *Chlorella protothecoides*, *Galdieria sulphuraria*, *Nitzschia laevis*, *Crypthecodinium cohnii,* and *Neochloris oleoabundans*, have been reported to have the potential to accumulate high biomass, fatty acids, or large quantities of valuable chemicals [4, 6–10].

Lipid accumulation in oleaginous microalgae depends on diverse factors, such as the strain in use, nutritional imbalances of medium components, the available carbon (C) and nitrogen (N) source, trophic mode (autotrophy, mixotrophy, or heterotrophy), cultivation conditions, and culture time [1, 11]. Multiple strategies have been proposed for lipid production improvement in various microorganisms. Among them, N limitation has been broadly reported as an effective approach for overproduction of storage lipids in oleaginous microalgal species [12]. In heterotrophic cultures, organic carbon sources are used, and carbon/nitrogen (C/N) ratio controls the switch between protein and lipid synthesis; thus, the C/N ratio of culture media is one of the most critical nutritional factors affecting lipid content [13]. It was reported that the initial C/N ratio must be greater than 20 for maximum lipid production by oleaginous microorganisms [14]. For the oleaginous yeast *Rhodosporidium toruloides*, lipid accumulation was observed at a C/N ratio of 30 and increased with a C/N ratio up to 120 using glucose as carbon source [15]. For *Chlorella sorokiniana*, it was shown that a C/N ratio of approximately 20 indicated a change from carbon to nitrogen limitation, and lipid content was increased at C/N ratio higher or lower than the critical value [16]. The C/N ratio depends not only on the strain of microorganisms but also on the medium composition in the cultivation, and types of carbon and nitrogen sources [17, 18].

*Tetradesmus bernardii*, a newly isolated high-yielding oleaginous microalgal strain when cultured under photoautotrophic conditions, is able to grow under heterotrophic conditions [19]. This study was aimed to investigate whether *T. bernardii* could accumulate a high lipid content in heterotrophic culture, and how to improve the lipid production of *T. bernardii* in heterotrophic culture. We determined the biomass and lipid accumulation dynamics under different C/N ratios at different glucose concentrations with an organic nitrogen source (urea) and inorganic nitrogen source (NaNO_3_). We also investigated the relationship of C, N uptake rates, and cellular C, N contents with the initial C/N ratio in the medium under two nitrogen sources. To further understand the metabolic mechanism of *T. bernardii* in heterotrophic culture, we conducted time-resolved transcriptome analysis under different C/N ratios.

## Results and discussion

### Effect of different glucose concentrations on the growth and lipid accumulation of *Tetradesmus bernardii* under two nitrogen sources with a nitrogen concentration of 18 mM

**Fig. 1:**
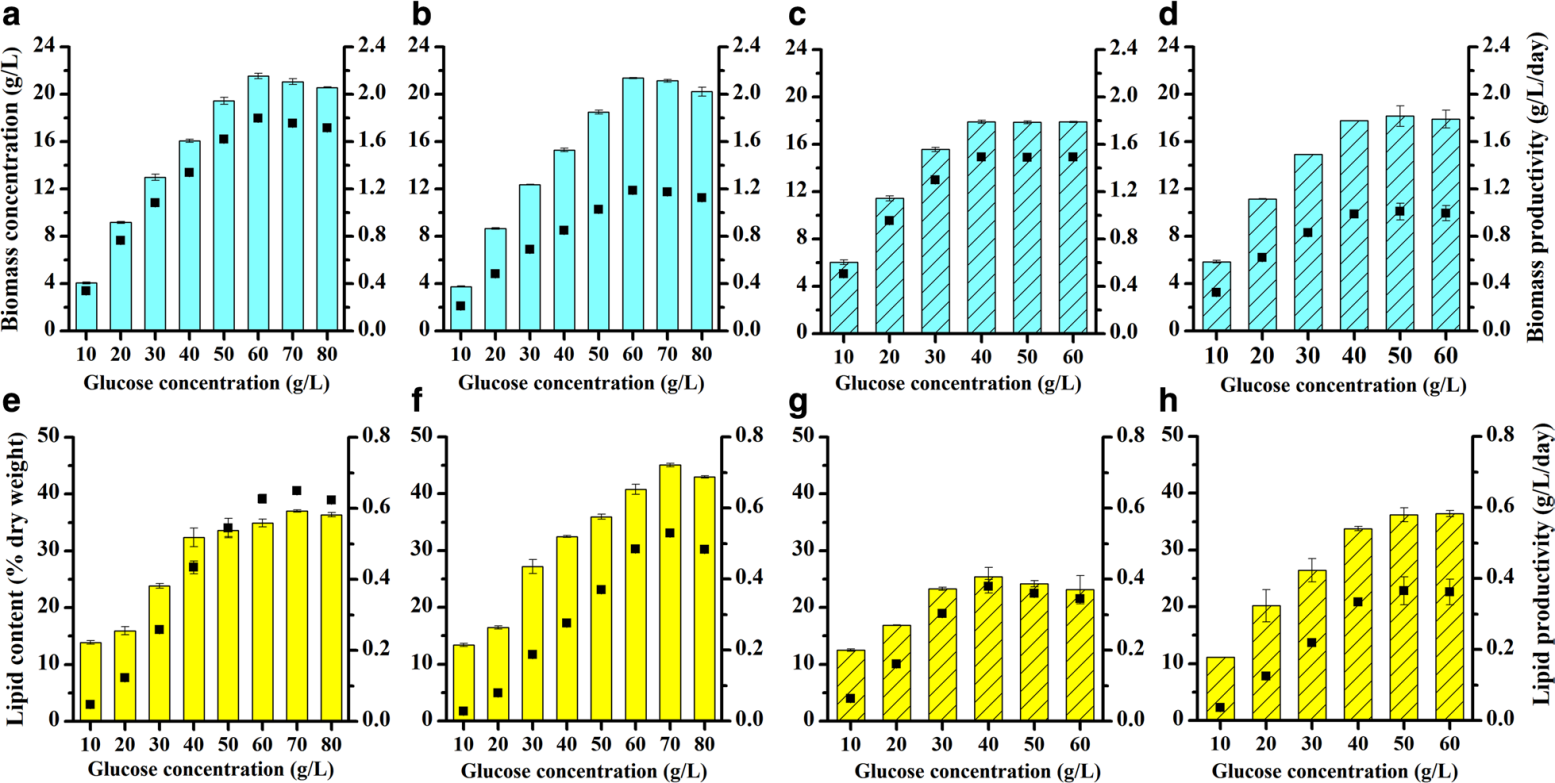
Effect of different glucose concentrations on the biomass concentration and biomass productivity (**a**–**d**), lipid content, and lipid productivity (**e**–**h**) of *T. bernardii*. Column represents biomass concentration (**a**–**d**) or lipid content (**e**–**h**). Point represents biomass productivity (**a**–**d**) or lipid productivity (**e**–**h**); **a**, **e** NaNO_3_ treatment, 12 days; **b**, **f** NaNO_3_ treatment, 18 days; **c**, **g** urea treatment, 12 days; **d**, **h** urea treatment, 18 days

Carbon is the main component in the microalgal cell and accounts for 17.5–65% of dry weight depending on the species and culture conditions [20]. In heterotrophic culture, glucose is a preferred carbon source to sustain growth for most microalgae, as it contains higher energy content than other organic substrates [21, 22]. To evaluate the effect of different glucose concentrations on growth and lipid accumulation, *T. bernardii* was cultured in mEndo medium with a glucose concentration ranging from 10 to 80 g/L and nitrogen concentration of 18 mM under inorganic and organic nitrogen sources, which were NaNO_3_ and urea, respectively. Biomass under different glucose concentrations showed that there was no significant difference in biomass on day 12 and day 18 (*P* > 0.05) (Fig. 1a–d). In the culture with NaNO_3_ used as nitrogen source and glucose concentration from 10 to 60 g/L, the biomass of *T. bernardii* increased with increasing initial glucose concentrations, and the highest biomass achieved was 21.55 g/L. When the glucose concentration was more than 60 g/L, biomass no longer increased but decreased. The decrease in biomass was likely due to high glucose inhibition, which is a common phenomenon in heterotrophic batch cultures [13]. The highest glucose-to-biomass conversion rate was 43.4%, which was obtained at a glucose concentration of 20 g/L. Biomass productivity was calculated based on biomass concentration and culture time, and its variation trend at different glucose concentrations was consistent with that of the biomass concentration. The maximum biomass productivity was achieved at glucose concentration of 60 g/L, which was 1.80 g/L/day at day 12 and 1.18 g/L/day at day 18. When urea was used as nitrogen source, the biomass of *T. bernardii* increased with increasing glucose concentrations from 10 to 40 g/L. There was no significant difference in biomass when the glucose concentration exceeded 40 g/L (*P* > 0.05). The highest biomass and biomass productivity after 12 days of culture was 17.90 g/L and 1.49 g/L/day, respectively. The conversion rates of glucose to biomass in this treatment were 55.4%, 54.65%, 50.23%, 43.5%, 34.72%, and 28.98% at the glucose concentrations of 10, 20, 30, 40, 50, and 60 g/L, respectively. Compared with biomass under two nitrogen sources, the biomass under heterotrophic culture with urea treatment was significantly higher than that of NaNO_3_ treatment when the glucose concentration was 10–40 g/L. The biomass of culture with NaNO_3_ treatment, however, was significantly higher than that of urea treatment when the glucose concentration was more than 50 g/L. The biomass of *T. bernardii* not only was affected by the initial glucose concentration but also varied with nitrogen sources.

In the culture with NaNO_3_ concentration of 18 mM, there was no significant difference between the lipid content of *T. bernardii* at day 18 and day 12 at the glucose concentrations of 10 and 20 g/L (Fig. 1e, f). When the glucose concentration was more than 30 g/L, the lipid content of *T. bernardii* at day 18 was significantly higher than that at day 12 (*P* < 0.01). The lipid content at day 18 showed an increasing effect of glucose concentration on lipid accumulation as glucose concentration increased from 10 to 70 g/L, and the highest lipid content reached 46.06% of dry weight. When the glucose concentration was more than 70 g/L, the lipid content decreased. Lipid productivity was a suitable evaluation parameter for lipid production which was calculated based on lipid content, biomass concentration, and culture time. Variation trend of lipid productivity was similar to the changes of biomass concentration and lipid content, which was increased firstly, but then decreased. The highest lipid productivity of 0.65 g/L/day was attained with the 70 g/L glucose concentration treatment at day 12. In the culture with urea used as nitrogen source and the nitrogen concentration of 18 mM, there was no significant difference between the lipid content of *T. bernardii* at day 18 and day 12 at the glucose concentration of 10 g/L. When the glucose concentration was more than 20 g/L, the lipid content of *T. bernardii* at day 18 was significantly higher than that of day 12. Lipid content and lipid productivity increased with glucose concentration from 10 to 40 g/L, and the maximum lipid content was 32.27% of dry weight at day 18, and the the maximum lipid productivity was 0.38 g/L/day at day 18. When the glucose concentration exceeded 40 g/L, the lipid content and lipid productivity no longer increased (Fig. 1g, h). In photoautotrophic culture, stress conditions commonly increase lipid content but decrease the biomass, whereas heterotrophic culture simultaneously accumulates lipids and biomass [13]. It was verified in *T. bernardii* cultured heterotrophically in both NaNO_3_ and urea as a nitrogen source. High lipid content and high biomass were both obtained at high glucose concentrations (70 g/L of glucose at NaNO_3_ group and 60 g/L of glucose at urea group). Notably, the effect of inorganic nitrogen source, NaNO_3_, on the increase of lipid content, was much better than that of organic nitrogen source, urea. Similar results were found in *Crypthecodinium cohnii*, where NaNO_3_ supported much higher lipid content (26.9% of dry weight) than other nitrogen sources, including urea [23]. In *Scenedesmus*, NaNO_3_ was also reported to yield the highest lipid content among other nitrogen sources [24]. In addition to the glucose concentration and nitrogen source, culture time also had a significant effect on lipid accumulation. Ordinarily, lipid content attained its maximum value at stationary growth phase in heterotrophic culture, and the cells should be harvested at early stationary phase because of lipid degradation [18]. In heterotrophic culture of *T. bernardii*, the cell reached stationary growth phase on day 12, while the lipid content on day 18 was much higher than it was on day 12 at high glucose concentration. Lipid accumulation could increase with the extension of culture time, which indicated that lipid synthesis was still active at the stationary phase in *T. bernardii*.

### Effects of different NaNO_3_ concentrations on the growth and lipid accumulation of *T. bernardii* at a glucose concentration of 60 g/L

**Fig. 2:**
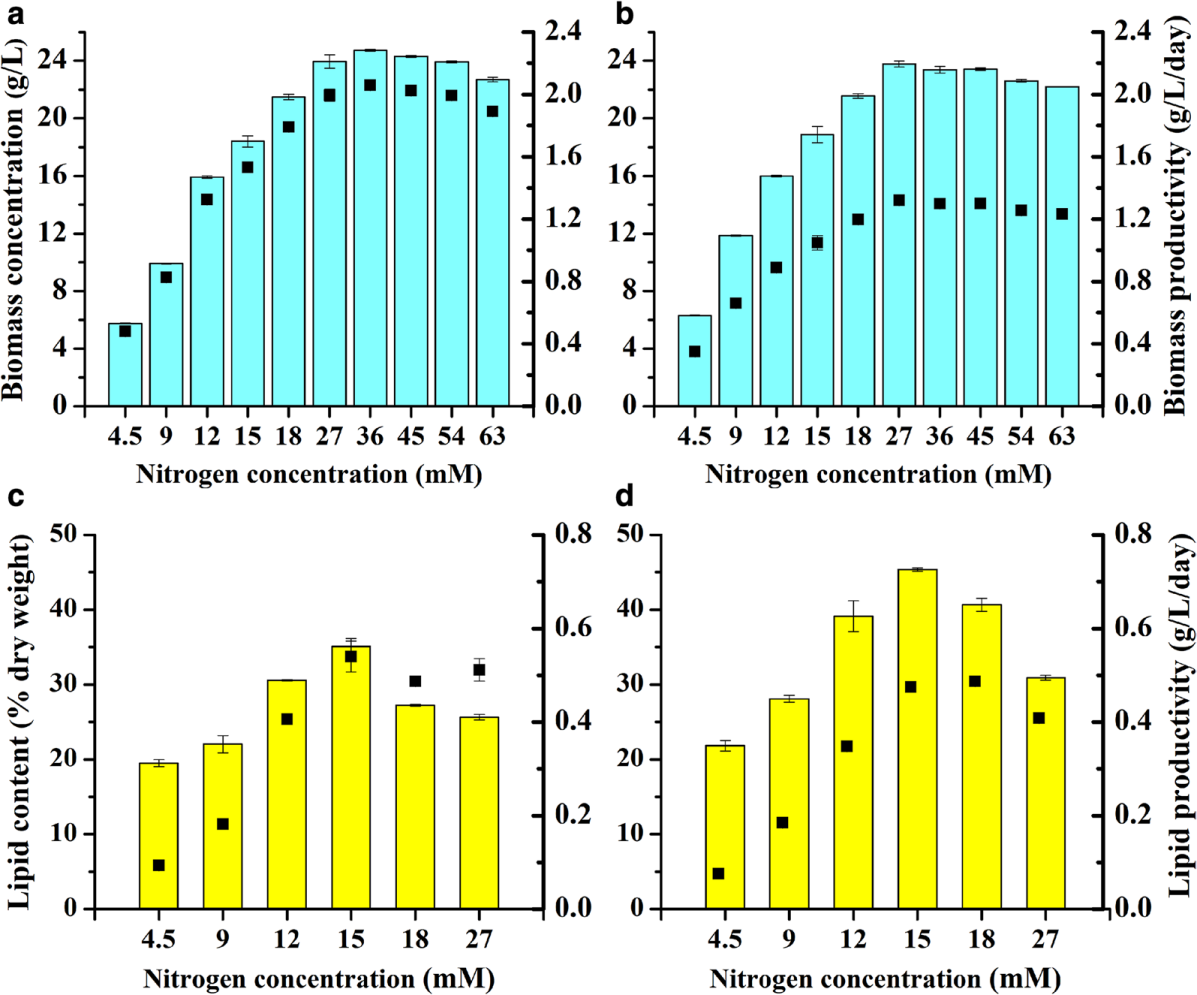
Effect of different NaNO_3_ concentrations on the biomass concentration and biomass productivity (**a**, **b**), lipid content, and lipid productivity (**c**, **d**) of *T. bernardii* at a glucose concentration of 60 g/L.Column represents biomass concentration (**a**, **b**) or lipid content (**c**, **d**). Point represents biomass productivity (**a**, **b**) or lipid productivity (**c**, **d**); **a**, **c** day 12; **b**, **d** day 18

According to the result that *T. bernardii* reached the highest biomass at a glucose concentration of 60 g/L, we investigated the effects of different NaNO_3_ concentrations on the biomass, lipid content, and lipid productivity of *T. bernardii* at the glucose concentration of 60 g/L (Fig. 2). When the nitrogen concentration of NaNO_3_ increased from 4.5 to 36 mM, the biomass concentration and biomass productivity of *T. bernardii* increased with the nitrogen concentration, and the highest biomass of 24.72 g/L and the highest biomass productivity of 2.06 g/L/day were achieved, with the highest glucose-to-biomass conversion rate of 40.37%. When nitrogen concentration was more than 36 mM, the biomass concentration and biomass productivity decreased with increasing nitrogen concentration. The lipid content of *T. bernardii* at day 18 was significantly higher than that at day 12 under different nitrogen concentrations. When the nitrogen concentration was 4.5–15 mM, the lipid content increased with an increasing nitrogen concentration. The lipid content decreased, however, with the increase of nitrogen concentration when the nitrogen concentration was above 15 mM. The highest lipid content of 45.35% of dry weight was obtained with a C/N molar ratio of 133, which was most suitable for lipid accumulation with NaNO_3_ used as nitrogen source. Meanwhile, this culture condition of C/N molar ratio reached the maximum lipid productivity, which is 0.54 g/L/day at day 12 of NaNO_3_ treatment. Compared to previous study of *T. bernardii*, with the highest lipid content of more than 65% of dry weight obtained under photoautrophic culture [19], the highest lipid content of 45% of dry weight under heterotrophic culture was much lower. This change of lipid accumulation caused by trophic mode was also observed in many other microalgae, such as *Chlorella vulgaris* UTEX 259, which accumulated lipid content of 38% of dry weight under photoautotrophic culture, and 23% of dry weight under heterotrophic culture [25]. However, there were some microalgae which accumulated much higher lipid content under heterotrophic culture than it under photoautotrophic culture, such as *Chlorella protothecoides.* The lipid content of *Chlorella protothecoides* obtained 14.57% of dry weight under photoautrophic culture, and it reached to 55.20% of dry weight under heterotrophic culture [26].

### Effects of different initial C/N ratios on the growth and lipid accumulation of *T. bernardii* under the culture with urea used as nitrogen source

We noticed that the conversion rates of glucose to biomass with urea used as nitrogen source was much higher than it with NaNO_3_, and the highest conversion rate reached to 55.4%. To identify the highest biomass when the culture with urea used as nitrogen source could be achieved, the culture with varying glucose concentrations (20, 40, 60, 80, 100, and 120 g/L) and different nitrogen concentrations of urea (3 to 120 mM) in *T. bernardii* was conducted. There was no significant difference in biomass between day 12 and day 18 at glucose concentrations of 20, 40, 60, 80, and 100 g/L (Additional file 1: Figures S1, S2). Biomass had a similar trend with the change in nitrogen concentration as that under different glucose concentrations, where biomass increased with increasing nitrogen concentration but then plateaued. At the glucose concentration of 20 g/L, the biomass of *T. bernardii* increased with enhancing nitrogen concentration from 3 to 18 mM, and the highest biomass of 10.87 g/L was obtained (Additional file 1: Figure S1a, b). When the nitrogen concentration was more than 18 mM, biomass decreased with the increase of nitrogen concentration. At the glucose concentration of 40 g/L and nitrogen concentration increased from 4.5 to 36 mM, the biomass of *T. bernardii* increased with nitrogen concentration (Additional file 1: Figure S1c, d). The highest biomass obtained was 23.07 g/L, and the glucose-to-biomass conversion rate was 56.43%. At the glucose concentration of 60 g/L, the biomass of *T. bernardii* increased with the increase of nitrogen concentration from 4.5 mM to 45 mM (Additional file 1: Figures S1e, f). The highest biomass was 34.48 g/L, and the glucose conversion rate was 56.63%. At the glucose concentration of 80 g/L and the nitrogen concentration of 4.5–60 mM, the highest biomass was 44.99 g/L, with glucose conversion rate of 55.61% (Additional file 1: Figures S2a, b). When the glucose concentration was 100 g/L and the nitrogen concentration of urea was 22.5–75 mM, the biomass of *T. bernardii* increased gradually, and the highest biomass was 46.09 g/L at which the glucose-to-biomass conversion rate was 45.59% (Additional file 1: Figures S1c, d). At the glucose concentration of 120 g/L, it was markedly different from that the glucose concentration of 20–100 g/L in heterotrophic culture (Additional file 1: Figure S2e, f). At all nitrogen concentrations, the biomass at day 12 was significantly different to that at day 18, and the highest biomass was only 24.13 g/L, at which the glucose conversion rate was 20%. This indicated that the glucose concentration of 120 g/L had a significant inhibitory effect on the growth of *T. bernardii*. Although glucose is a common organic carbon source in heterotrophic culture, the specific effects of glucose on the metabolism of microalgae vary greatly with species. For *Chlorella vulgaris*, due to the glucose absorption, the hexose/H^+^ transport system alkalinized the medium when there was sufficient glucose [27]. Thus, the high levels of glucose have been shown to inhibit microalgal growth, at least for a while. In order to promote cell growth of *C. vulgaris* and *Scenedesmus acutus*, initial glucose concentrations should be limited to 10 g/L and 1 g/L, respectively [28]. For *C. saccharophila*, the glucose concentration for optimal growth rate was 2.5 g/L, and it would inhibit growth when glucose concentration exceeded 25 g/L. Inhibition of *C. sorokiniana* appeared at glucose concentration of 5 g/L [29]. *C. protothecoides* achieved the optimal yield at a glucose concentration of 85 g/L [30]. In *Nitzschia laevis*, biomass yield gradually decreased with the increase of glucose concentration from 1 to 40 g/L [31]. *G. sulphuraria* can grow at a glucose concentration of 166 g/L, but their growth was inhibited at a high glucose concentration [32]. The highest biomass of *T. bernardii* was obtained at glucose concentration of 100 g/L, but the conversion rate of glucose to biomass at 100 g/L glucose was lower than it at glucose concentration of 80 g/L, which indicated that high glucose concentration of 100 g/L had a certain degree of inhibition on the growth of *T. bernardii*, but *T. bernardii* could still obtain high biomass concentration at a high glucose concentration of 100 g/L, which demonstrated that *T. bernardii* had a relatively high glucose tolerance. This inhibition on the growth of *T. bernardii* was even more pronounced at a glucose concentration of 120 g/L when biomass was greatly decreased. An appropriate glucose concentration for microalgal growth in heterotrophic culture may be related to a variety of factors, of which microalgae species is the main factor, followed by the culture conditions [3].

**Fig. 3:**
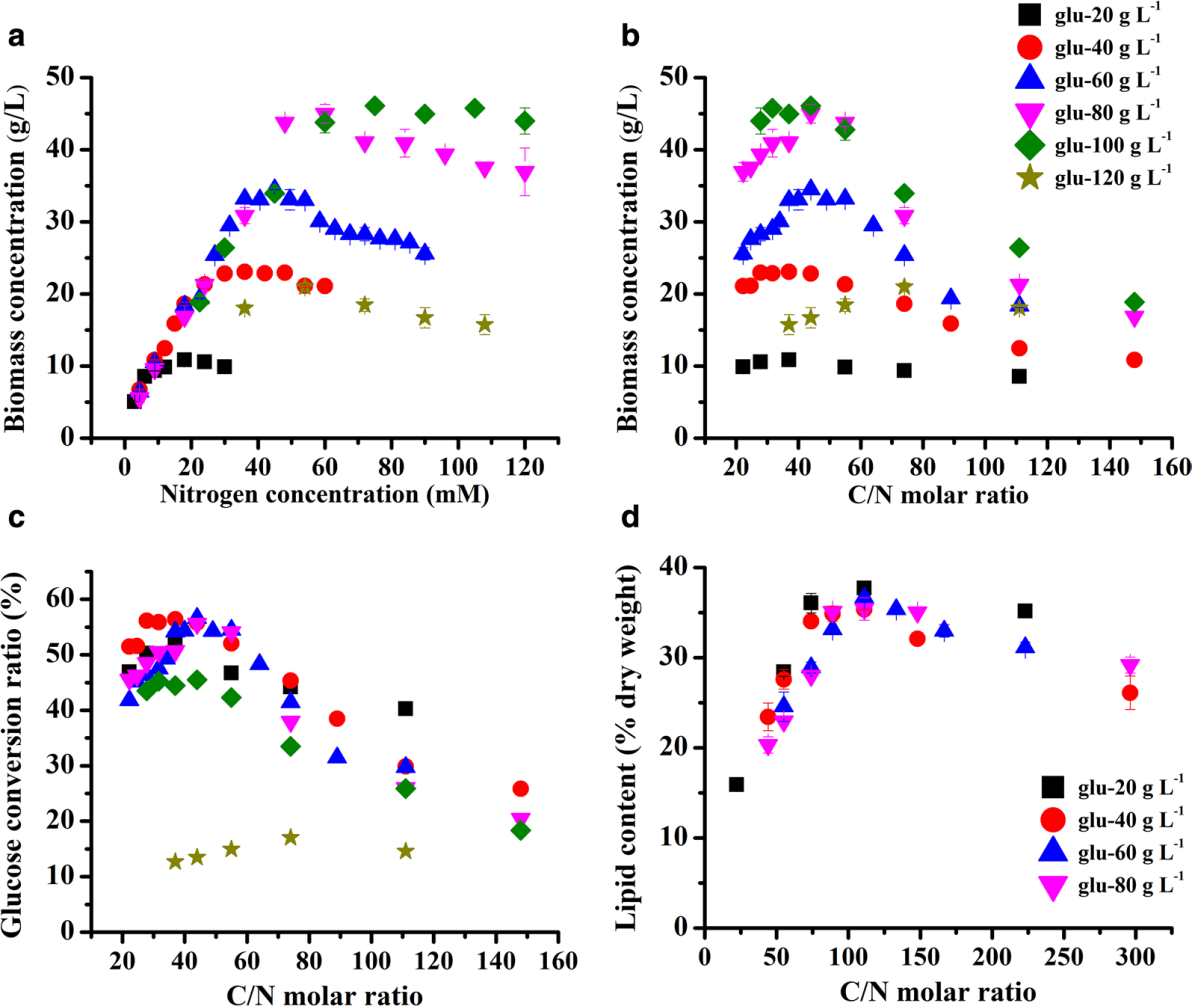
Effect of different culture conditions on the biomass (**a**, **b**), glucose-to-biomass conversion ratio (**c**), and lipid content (**d**) of *T. bernardii*

The biomass on day 12 of the above culture is summarized and presented in Fig. 3a. The biomass of *T. bernardii* in heterotrophic culture was closely related to nitrogen concentration and glucose concentration. At a relatively low nitrogen concentration, the biomass was mainly determined by nitrogen concentration. For instance, at a nitrogen concentration of 9 mM, the biomass was about 10 g/L, regardless of whether the initial glucose concentration was 20, 40, 60, or 80 g/L. Furthermore, the correlation between nitrogen concentration and biomass revealed a strong linear dependence. When the glucose concentration was 40, 60, and 80 g/L and the nitrogen concentration was less than 48 mM, the relationship between nitrogen concentration and biomass was *y* (biomass) = 0.834*x* (nitrogen concentration) + 2.50 (*R*^2^ = 0.991, *P* < 0.01). Specifically, at the glucose concentration of 40 g/L and nitrogen concentration ≤ 24 mM, it was *y* = 0.772*x* + 3.69 (*R*^2^ = 0.981, *P* < 0.01). There were *y* = 0.841*x* + 2.54 (*R*^2^ = 0.991, *P* < 0.01) (nitrogen concentration ≤ 36 mM) and *y* = 0.857*x* + 1.41 (*R*^2^ = 0.996, *P* < 0.01) (nitrogen concentration ≤ 48 mM), which were at the glucose concentrations of 60 and 80 g/L, respectively. When the glucose concentration was 100 g/L and nitrogen concentration was less than 75 mM, the relationship between nitrogen concentration and biomass was *y* = 0.523*x* + 9.49 (*R*^2^ = 0.955, *P* < 0.01). At this time, the adjusted *R*^2^ (0.955) was lower than that at the glucose concentrations of 40, 60, and 80 g/L, indicating that the linear relationship between biomass and nitrogen concentration was inhibited to some extent by the high glucose concentration. Safdar et al. [23] also reported that the growth of *Crypthecodinium cohnii* in heterotrophic culture was linearly correlated with N supply to a certain level and then showed a negative impact. At the glucose concentration of 120 g/L in *T. bernardii*, the biomass was significantly affected by the high glucose concentration. Meanwhile, at a high nitrogen concentration, the biomass of *T. bernardii* was mainly determined by glucose concentration. For example, at the glucose concentration of 20 g/L, the highest biomass was 10.87 g/L, and at the glucose concentration of 80 g/L, the highest biomass was 44.99 g/L.

The carbon molar concentration of glucose per gram was 33 mM, so the relationship between C/N ratio and biomass was calculated (Fig. 3b). A high or low C/N ratio in the medium was not conducive to the accumulation of biomass and the biomass reached the maximum under an appropriate C/N. This was consistent with *Auxenochlorella protothecoides*, in which the highest dry weight was achieved at a C/N (g/g) ratio of 40 and the biomass decreased at both higher and lower C/N ratios [21]. By associating biomass with glucose concentration in the medium, the relationship between the conversion rate of glucose to biomass and C/N ratio in the medium was also calculated (Fig. 3c). When the glucose concentration was 20–80 g/L, the conversion rate of glucose to biomass reached more than 50% in the culture with a C/N ratio from 37 to 55, and when the C/N ratio was 44, the conversion rate of glucose to biomass exceeded 55%. The high conversion rate of glucose to biomass of *T. bernardii* showed its great potential for application in biomass production of heterotrophic culture.

At glucose concentrations of 20, 40, 60, and 80 g/L, the lipid content of *T. bernardii* in the heterotrophic culture with different urea concentrations was determined. The lipid content of *T. bernardii* at day 18 was much higher than the lipid content at day 12 (Additional file 1: Figures S3; S4). In culture with the glucose concentration of 20 g/L, and nitrogen concentration from 3 to 9 mM, the highest lipid content was 30% of the dry weight obtained at day 12. When the culture time was prolonged, the lipid content was increased. The highest lipid content was obtained at the nitrogen concentration of 6 mM, which was 37.72% of the dry weight (Additional file 1: Figure S3). At the glucose concentrations of 40, 60, and 80 g/L, the highest lipid content in *T. bernardii* after 18 days was 35.35, 36.66, and 35% of the dry weight, respectively, which was obtained at nitrogen concentrations of 12, 18, and 24 mM, respectively (Additional file 1: Figures S3c, d, S4a–d).

Lipid accumulation in *T. bernardii* under heterotrophic culture was closely related to C/N molar ratio (Fig. 3d). With an increase of the C/N ratio, the lipid content in the cells increased and then decreased. The C/N molar ratio of 110–130 was most beneficial to the accumulation of lipids in the heterotrophic culture of *T. bernardii*. This finding was in accordance with the report by Papanikolaou and Aggelis [14], in which the highest lipid production needs initial C/N ratio of greater than 20 in oleaginous microorganisms. The highest lipid concentration in *Auxenochlorella protothecoides* was observed at a C/N (g/g) ratio of 60 [21]. Reportedly, however, the lipid content in *C. sorokiniana* was lowest at a C/N ratio of approximately 20 and increased at higher or lower C/N values [16]. The response of microalgal lipid accumulation to C/N ratio varied with species. This may depend on the main carbon storage compounds of microalgae. As some microalgae preferred to accumulate lipid as main carbon storage compounds, some preferred to store carbohydrate.

The effect of C/N ratio on lipid productivity under urea treatment is presented in Additional file 1: Figure S5. The change of lipid productivity is similar to that of biomass concentration and lipid content. With an increase of the C/N ratio, the lipid productivity increased firstly, and then decreased. The culture condition that achieved the highest lipid productivity was mainly determined by the culture that obtained the highest biomass concentration (Additional file 1: Figure S5). Thus, the highest lipid productivity of 0.74 g/L/day was achieved at 100 g/L glucose concentration with C/N ratio of 55, and the C/N ratio of 55 has a high conversion rate of glucose to biomass reached more than 50%.

### Change in the growth, glucose uptake, and cellular C and N contents of *T. bernardii* in different culture conditions over time

**Fig. 4:**
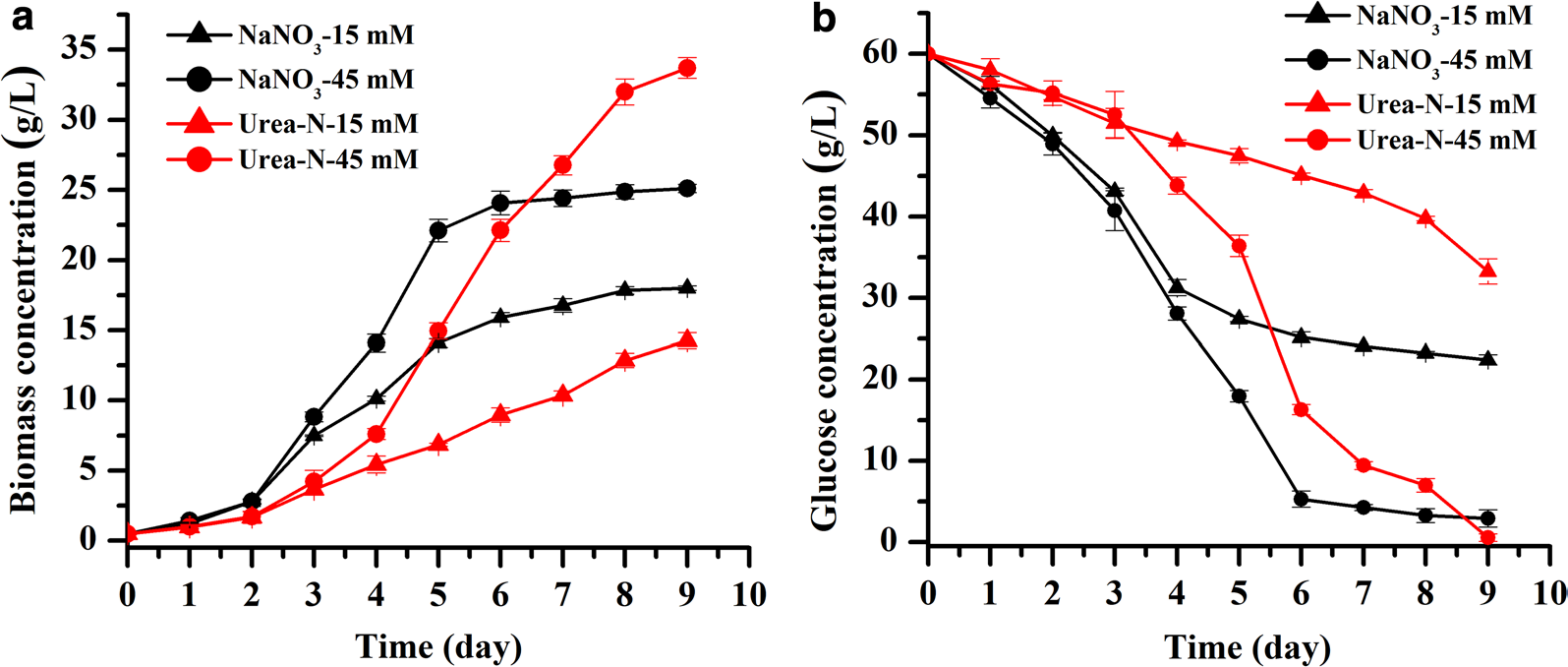
Time-course change of growth (**a**) and residual glucose concentration in the medium (**b**) at glucose concentration of 60 g/L

To further analyze the influence of NaNO_3_ and urea on the growth and lipid accumulation of *T. bernardii* in heterotrophic culture, time-course change of glucose and nitrogen uptake rate, and cellular C and N contents in the culture with nitrogen concentrations of 15 mM and 45 mM at glucose concentration of 60 g/L were investigated (Fig. 4a). When NaNO_3_ was used as nitrogen source, the growth rate of *T. bernardii* in the early phase of culture was much higher than that in the urea treatment. The growth rate at nitrogen concentration of 45 mM was higher than the nitrogen concentration of 15 mM during the exponential growth phase after the third day of culture. The treatment of 45 mM NaNO_3_ reached the stationary phase on the sixth day of culture. The final biomass was 25.1 g/L and the conversion rate of glucose to biomass was 41%. When urea was used as a nitrogen source and the nitrogen concentration was 45 mM, the biomass was higher than that of NaNO_3_ treatment after seven days of culture. The biomass was 33.71 g/L on the ninth day of cultivation, and the conversion rate of glucose to biomass was 55%. At low nitrogen concentration, the growth was always higher when NaNO_3_ used as nitrogen source rather than urea. Interestingly, the effect of NaNO_3_ and urea on biomass in *T. bernardii* was dependent on the nitrogen concentrations. At high nitrogen concentration, urea resulted in higher biomass accumulation, while at low nitrogen concentration, *T. bernardii* grew faster with NaNO_3_ than with urea. In *Scenedesmus bijugatus*, the highest specific growth rate was presented in the culture with NaNO_3_ and KNO_3_ and urea exhibited less of an effect on biomass [33]. A similar result was observed in *Crypthecodinium cohnii* [23]. Conversely, in *Chlorella protothecoides*, the highest yield of biomass was obtained in the culture with urea as nitrogen sources, when compared with nitrate and ammonium [30].

The concentration of residual glucose in the medium showed that the glucose uptake rate was closely correlated with growth (Fig. 4b). At the initial stage of culture, the glucose uptake rate in the culture with NaNO_3_ used as nitrogen source was much higher than that of the urea treatment. In the middle and late stages of culture, the glucose uptake rate of the high nitrogen concentration (45 mM) was higher than that of the low nitrogen concentration (15 mM). There was almost no glucose remaining in the high nitrogen concentration treatment. At low nitrogen concentration, there were 37% and 55% of the original glucose concentrations remaining in the culture of NaNO_3_ and urea treatment, respectively. It was indicated that NaNO_3_ used as nitrogen source was more conducive to the rapid absorption of glucose by *T. bernardii* compared with the urea treatment under the same nitrogen concentration. Under the high nitrogen concentration, the biomass of the urea treatment was much higher than that of the NaNO_3_ treatment. Urea used as nitrogen source was more beneficial to the transformation of glucose into biomass by *T. bernardii* meaning high conversion rate of glucose to biomass.

The analysis of cellular C and N contents is presented in Table 1. The N absorption rate of the cells was calculated by the cellular N content combined with the biomass concentration. In the culture with NaNO_3_ used as nitrogen source, the cellular N content on the first day of culture was higher than that of the urea treatment. The absorption rate of NaNO_3_-N in the early stage of heterotrophic culture was higher than the rate of urea-N uptake by *T. bernardii*. With the prolongation of culture time, the cellular N content decreased and the cellular C content increased when NaNO_3_ was used as nitrogen source, so the cellular C/N ratio increased gradually. On the last day of culture, 15 mM NaNO_3_ was absorbed completely by *T. bernardii*, but only 80% of total nitrogen from 45 mM NaNO_3_ was absorbed. When urea was used as nitrogen source, cellular N content increased with culture time and then decreased. The cellular C content increased gradually, and cellular C/N ratio decreased and then increased. On the last day of culture, *T. bernardii* completely absorbed 15 and 45 mM of urea-N. Nitrate is a major source of nitrogen that has a strong influence on microalgal growth and metabolism [1]. Nitrate assimilation was influenced by environment, and most microalgae assimilate nitrate more rapidly in the light than in the dark [1]. Our previous study confirmed that the uptake of NaNO_3_ in *T. bernardii* was faster in phototrophic culture than in heterotrophic culture [19]. In this study, we noticed that NaNO_3_ assimilation was faster than urea assimilation in the early stage of culture in *T. bernardii.* Combined with higher glucose uptake rate in NaNO_3_ treatment, it resulted in higher growth rate of *T. bernardii* in the culture with NaNO_3_ used as nitrogen source than in the culture with urea. At the late stage of culture, high concentration of NaNO_3_ (45 mM) and glucose was not absorbed completely by *T. bernardii*, and high concentration of urea and glucose was absorbed completely, and thus, the biomass at high concentration of NaNO_3_ was lower than that achieved in the urea treatment. The cellular C/N ratio showed that the value at low concentration of NaNO_3_ treatment was higher than that in the urea treatment, which may lead to high lipid accumulation.

**Table 1:** Cellular C and N contents of *T. bernardii* under herterotrophic culture

		Day 1	Day 2	Day 3	Day 4	Day 5	Day 6	Day 7	Day 8	Day 9
NaNO_3_- 15 mM	C	44.38 ± 0.54	45.05 ± 0.27	46.41 ± 0.34	47.26 ± 0.21	48.73 ± 0.17	50.09 ± 0.20	50.61 ± 0.15	51.16 ± 0.22	52.51 ± 0.31
	N	4.66 ± 0.24	3.62 ± 0.31	2.24 ± 0.27	1.35 ± 0.42	1.51 ± 0.19	1.13 ± 0.07	1.17 ± 0.10	1.14 ± 0.06	1.19 ± 0.12
	C/N	9.53	12.43	20.75	34.93	32.22	44.19	43.15	44.88	44.12
NaNO_3_- 45 mM	C	44.63 ± 0.27	45.14 ± 0.33	46.15 ± 0.41	46.76 ± 0.18	47.38 ± 0.35	48.5 ± 0.22	49.34 ± 0.18	49.68 ± 0.19	50.39 ± 0.25
	N	4.93 ± 0.12	4.60 ± 0.24	4.32 ± 0.31	4.30 ± 0.43	2.56 ± 0.41	2.36 ± 0.17	2.05 ± 0.11	2.09 ± 0.16	2.09 ± 0.20
	C/N	9.06	9.82	10.69	10.87	18.5	20.59	24.04	23.77	24.11
Urea-N- 15 mM	C	41.92 ± 0.36	44.26 ± 0.28	46.29 ± 0.54	45.67 ± 0.17	45.41 ± 0.21	46.46 ± 0.22	46.99 ± 0.19	47.66 ± 0.24	48.54 ± 0.36
	N	3.19 ± 0.24	4.23 ± 0.31	5.60 ± 0.43	3.83 ± 0.26	2.94 ± 0.17	2.07 ± 0.22	1.93 ± 0.14	1.35 ± 0.08	1.27 ± 0.10
	C/N	13.12	10.46	8.26	11.93	15.42	22.46	24.40	35.30	38.22
Urea-N- 45 mM	C	44.74 ± 0.24	44.50 ± 0.18	45.51 ± 0.32	46.15 ± 0.41	46.47 ± 0.37	47.05 ± 0.31	47.74 ± 0.44	48.07 ± 0.28	48.37 ± 0.26
	N	3.39 ± 0.21	4.29 ± 0.34	5.29 ± 0.37	5.78 ± 0.18	3.62 ± 0.21	2.52 ± 0.16	2.14 ± 0.10	1.81 ± 0.11	1.61 ± 0.09
	C/N	13.20	10.37	8.60	7.99	12.85	18.70	22.32	26.56	30.02

### Transcriptome analysis of the *T. bernardii* under heterotrophic culture

In order to elucidate the metabolic mechanism of *T. bernardii* in heterotrophic culture, time-resolved transcriptome analysis under different C/N ratios was conducted. The heterotrophic culture of *T. bernardii* at glucose of 40 g/L with urea-N concentrations of 9 mM and 27 mM was performed, and then samples were collected at 0 h, 12 h, 2 d, 3 d, 5 d, 9 d, 12 d, and 15 d for the measurements of RNA-seq data.

**Fig. 5:**
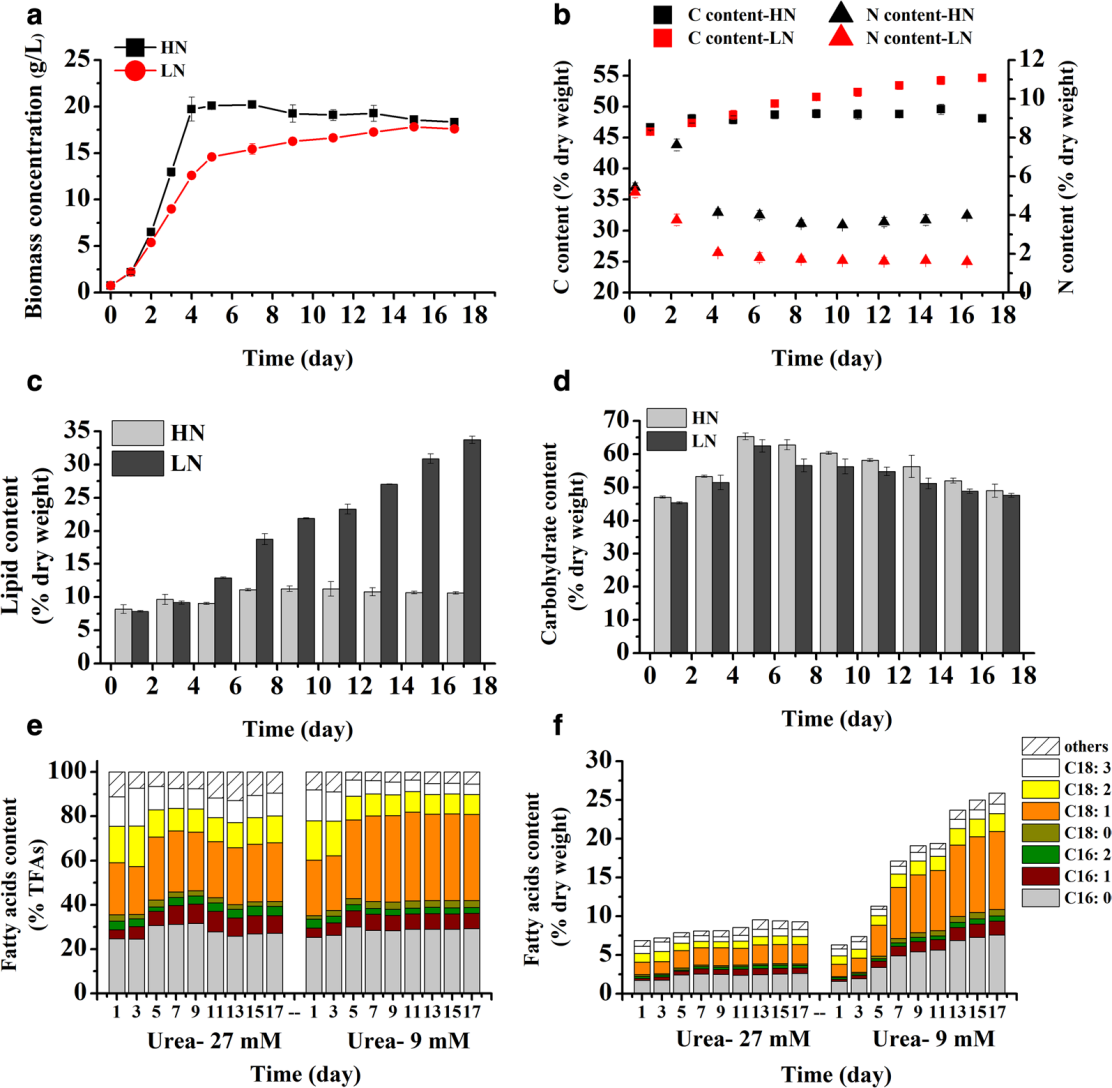
Time-course change of growth and biochemical composition of *T. bernardii* under heterotrophic culture

Under a high N concentration condition (27 mM, HN), the growth rate of *T. bernardii* was higher than under a low nitrogen concentration culture (9 mM, LN), and it reached the stationary phase on the fifth day of culture, in which the highest biomass of 20.22 g/L was achieved (Fig. 5a). The biomass increased gradually under LN and it reached 17.60 g/L. The cellular C content of *T. bernardii* increased with culture time, but the range of increase varied with nitrogen concentration (Fig. 5b). Cellular C content under LN was higher than it in HN culture after the seventh day. The content of cellular N under HN increased and then decreased, with 3.5–4% of dry weight remaining; the variation range of the cellular C/N ratio was smaller compared with LN, in which cellular N content decreased to 1.6% of dry weight. In HN culture, the lipid content of *T. bernardii* was about 10% of dry weight, whereas in LN, the lipid content increased gradually with culture time and the lipid content reached 34% of dry weight (Fig. 5c). The carbohydrate content of *T. bernardii* was more than 45% of dry weight in the whole culture time (Fig. 5d). It peaked at 65% and 62% of dry weight on the fifth day under HN and LN, respectively. We noticed that *T. bernardii* accumulated a high content of carbohydrate in heterotrophic culture, which was used as primary carbon storage compound. The fatty acid composition of *T. bernardii* was mainly palmitic acid (C16:0), oleic acid (C18:1), linoleic acid (C18:2), and linolenic acid (C18:3), which accounted for more than 80% of the total fatty acid content (Fig. 5e). Under LN, the content of C18:1 increased 1.6 times which made up 40% of the total fatty acids on day 17, and the content of C18:2 and C18:3 was decreased. Under HN, the change in fatty acid content of total fatty acids was relatively small, and the total fatty acid content of dry weight had no significant increase over culture time ( 7% to 9% of dry weight) (Fig. 5f). Under LN, the total fatty acid content increased gradually, and reached 26% of dry weight.

For reference transcriptome analysis, we collected 15 samples at different time points mentioned above in HN and LN culture and extracted total RNA. Illumina Hiseq 4000 platform was used for double-ended sequencing, and a total of 11.01 Gb of clean reads were obtained (Additional file 2: Table S1). The number of unigenes after assembly was 40,697 with a GC content of 55.64%. For functional annotation, a total of 25,062 unigenes (61.58%) were annotated (Additional file 1: Figure S6), of which the number of unigenes annotated by the NR database was the most (21,634), accounting for 53.16%. Unigenes annotated by NR database matched at least one species. The species distribution of the NR annotation of *T. bernardii* was statistically analyzed (Additional file 1: Figure S7). It was found that *Volvox carteri* f. *nagariensis* had the highest matching degree with the *T. bernardii* at 23.17% (5,012). There were 3809 genes in *T. bernardii* matched with *Chlamydomonas reinhardtii* and, 2,214 and 1,890 genes were matched with *Chlorella variabilis* and *Coccomyxa subellipsoidea* c-169, respectively. These four species of microalgae belong to the same family and same class of chlorophyta, which indicated that the results of this gene annotation had very high reliability.

To investigate the response and adaptation of *T. bernardii* to different C/N ratios in heterotrophic culture, the cells grown under HN and LN were collected at 0 h, 12 h, 2 d, 3 d, 5 d, 9 d, 12 d, and 15 d in culture, and RNA-seq for each sample was conducted. RSEM was used to calculate the expression of each sample, and the different expression genes (DEGs) in each sample were compared with 0 h as the control (Additional file 1: Figure S8). A total of 9,100 genes were up-regulated and 15,690 genes were down-regulated under HN. Under LN, a total of 10,058 genes were up-regulated and 9964 genes were down-regulated. With the extension of culture time, the nutrients in the culture medium were consumed, and a large number of genes were down-regulated under stationary phase. Enrichment analysis of KEGG metabolic pathways was carried out for all up-regulated and down-regulated genes under HN and LN. The top 20 metabolic pathways that were significantly up-regulated and down-regulated are presented in Additional file 1: Figure S9. Under HN, with the consumption of nutrients in the culture medium, the genes associated with growth, such as ATP-binding cassette (ABC) transporters that hydrolyze ATP to transport sugar, lipids, and peptides, were significantly down-regulated. There were 39 unigenes encoding ABC transporters which were down-regulated. The related genes of RNA transport and carbon fixation (22 unigenes) were down-regulated. Meanwhile, the related gene of fatty acid elongation, trans-2-enoyl-CoA reductase (Unigene11017), 3-ketoacyl-CoA synthase (Unigene6926, CL562.Contig2, Unigene1412), very-long-chain 3-oxoacyl-CoA reductase (Unigene947), and very-long-chain 3-hydroxyacyl-CoA dehydratase (Unigene28343) were continuously down-regulated after the 9th day, resulting in low fatty acid content and no significant change in the content of long-chain unsaturated fatty acid. In addition, the related genes of carotenoid biosynthesis (17 unigenes) were significantly down-regulated. The up-regulated genes in the LN were mainly enriched in proteasome, fatty acid metabolism, glycerophospholipid metabolism, and N metabolism. The down-regulated genes in the LN were mainly enriched in the pathway of photosynthesis–antenna proteins (ko00196), porphyrin and chlorophyll metabolism (ko00860), and photosynthesis (ko00195).

**Fig. 6:**
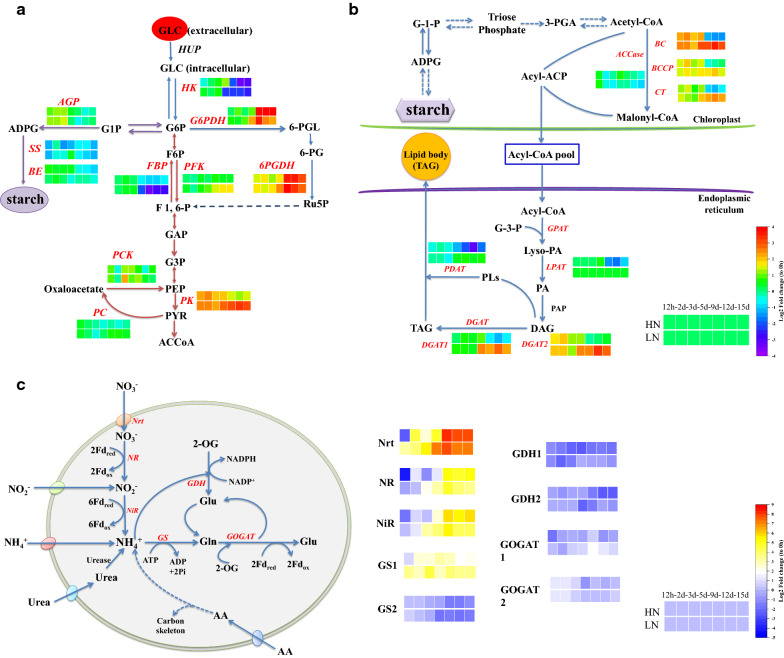
Central carbon metabolism (**a**, **b**) and nitrogen metabolism (**c**) in *T. bernardii* under heterotrophic culture

Fatty acid biosynthesis is often limited by the supply of NADPH, as the elongation of fatty acids during their biosynthesis by two carbon units requires two NADPH molecules [11]. Oxidative pentose phosphate pathway provides NADPH to the cell through the oxidation of glucose. Its key enzymes include glucose-6-phosphate dehydrogenase (G6PDH) and 6-phosphogluconate dehydrogenase (6PGDH). Under their co-catalysis, glucose-6-phosphate is oxidized to ribulose 5-phosphate (Ru5P) accompanied by the production of NAPDH. Under HN and LN, the expression of genes encoding G6PDH (Unigene3815) and 6PGDH (Unigene30058) did not change significantly in the early stage of culture, but were significantly up-regulated in the late stage of culture (Fig. 6a). This result suggested that with the extension of culture time, more NADPH is needed to synthesize storage substances.

Three key enzymes of the glycolysis pathway, including hexokinase (HK), 6-phosphofructokinase (PFK, rate-limiting enzyme), and pyruvate kinase (PK) [34], catalyzed the process which is irreversible, as well as the corresponding enzymes in the glucogenesis pathway, including glucose 6-phosphatase, fructose 1, 6-bisphosphatase (FBP), pyruvate carboxylase (PC), and phosphoenolpyruvate carboxykinase (PCK). At the early stage of heterotrophic culture, the gene encoding HK (Unigene18767) was up-regulated firstly, and then was down-regulated. This was consistent with the high glucose content obtained by cells that absorb glucose from the medium at the early stage of culture. In LN culture, the genes encoding PFK (Unigene24519) and PK (Unigene12413) were up-regulated with the culture time. Compared with HN, the variation range in LN was relatively small. This result indicated that the glycolysis pathway was more significant at the late stage in LN. The gene encoding FBP was down-regulated over culture time, and the expression changes of the genes encoding PC and PCK in the glucogenesis pathway were relatively moderate in HN and LN. Changes in their expression levels indicated the direction of the carbon flow within the cell.

ADP-glucose pyrophosphorylase (AGP) catalyzes the synthesis of ADP-glucose (ADPG) which is the precursor of starch synthesis. Starch synthase (SS) catalyzed the transfer of glucose from ADPG to the non-reductive end of α-1, 4-glucan. The branching enzyme (BE) catalyzed the formation of an α-1, 6-glycosidic bond. These three enzymes were the key enzymes for starch synthesis (Fig. 6a). The gene encoding AGP (Unigene27221) was up-regulated in the early period, and then was down-regulated, which was consistent with the changes in cellular carbohydrate content (Fig. 5). Not that genes in the gluconeogenic pathway were not significantly up-regulated, so glucose, the precursor for the synthesis of starch, was likely to be directly derived from the external organic carbon source absorbed by the cell.

Synthesis of fatty acids begins with acetyl-coenzyme A (CoA), which produces malonyl-CoA under the catalysis of acetyl-coenzyme A carboxylase (ACCase). ACCase is the first enzyme in the fatty acid synthesis pathway and is also a key rate-limiting enzyme [2]. In algae, ACCase has two different forms, a heteromeric form and a homomeric form [35]. It is generally believed that heteromeric ACCase, also known as prokaryotic ACCase, exists in plasmids, and homomeric ACCase, also known as eukaryotype ACCase, exists in the cytoplasm. Heteromeric ACCase has four functional subunits, including the biotin carboxyl carrier protein (BCCP), biotin carboxylase (BC), and α- and β-carboxyltransferases (CT). The gene of α-CT was encoded by the chloroplast genome, and the other three subunits were encoded by nuclear genes. Homomeric ACCase is a single homologous dimer protein; these four subunits are located on one polypeptide and it is encoded by the nuclear gene acc1, which is highly conserved. The change in gene expression of the three subunits encoding heteromeric ACCases in *T. bernardii* was consistent over time. The expression of genes encoding BCCP (Unigene18839), BC (Unigene8052), and CT (Unigene30882) were significantly up-regulated at the late stage in LN compared with those of HN. The change in the gene encoding homomeric ACCase (Unigene29953) was relatively moderate. This result indicated that the changes of ACCase were mainly regulated by the heteromeric form.

TAG synthesis includes the acyl-CoA-dependent pathway, which is also called the Kennedy pathway and acyl-CoA-independent pathway. For the Kennedy pathway, acyl-CoAs are sequentially added onto the sn-1, sn-2, and sn-3 positions of glycerol-3-phosphate by the catalyzation of glycero-3-phosphate acyltransferase (GPAT), lysophospholipid acyltransferase (LPAT), and diacylgycerol acyltransferase (DGAT), respectively (Fig. 6b). DGAT is the last and rate-limiting enzyme in TAG biosynthesis [2]. Two types of membrane-bound DGATs are known to have different protein sequences, functions, expression patterns, and organelle sites [36]. The acyl-CoA-independent pathway of TAG synthesis is catalyzed by phospholipid: diacylglcerol acyltransferase (PDAT). PDAT uses phospholipids as acyl donors and DAGs as the acyl receptors to produce TAGs. *C. reinhardtii* has five genes encoding type II DGATs, one gene encoding type I DGAT, and one PDAT gene. In *T. bernardii*, nine unigenes encode type 1 DGAT (Unigene22686, cl825. Contig1, Unigene23892, Unigene27837, cl1943.contig1, Unigene27236, Unigene3503, Unigene14224, and CL1495.Contig1), and two genes encode type I DGAT (Unigene10373 and Unigene34540) and two PDAT genes (one type I (Unigene30932) and one type II (Unigene28641)). The DGAT2 gene was up-regulated in the culture of *T. bernardii*, and its expression level was significantly higher than that of DGAT1, indicating that type II DGAT played a major role in the metabolic process. At the same time, the comparison between LN and HN showed that the genes related to lipid synthesis were significantly up-regulated at the late stage of culture in LN. Regarding fatty acid degradation, we found that the genes encoding triglyceride lipase were significantly up-regulated. This may have resulted in the slow rate of lipid accumulation under heterotrophic metabolism.

To investigate the effects of different C/N ratios on the nitrogen assimilation in *T. bernardii*, we analyzed the main nitrogen metabolism. The most commonly used nitrogen source in microalgae is nitrate. The nitrate ions (NO_3_^−^) are transported into microalgal cells by nitrate transporter proteins (Nrt). After entering cells, nitrate is reduced to ammonium through sequential reactions catalyzed by nitrate reductase (NR) and ferredoxin-nitrite reductase (NiR) [3]. The genes encoding Nrt, NR, and NiR in *T. bernardii* were significantly up-regulated with the extension of culture time (Fig. 6c). It indicated that with the consumption of nitrogen in the culture, the cells responded to the nitrogen stress by increasing the expression of the genes related to nitrogen uptake. Interestingly, *T. bernardii* used the urea as the nitrogen source, but related genes of urease were not significantly up-regulated, indicating that genes encoding urease were not regulated at the transcriptional level. Inorganic ammonia forms organic nitrogen compounds via the catalyzation by glutamate dehydrogenase (GDH) or glutamine synthetase (GS) to yield glutamate (Glu). Glu, as the most abundant amino acid in organisms, is the main nitrogen donor among nitrogenous compounds. GDH includes two types, NADPH dependent (GDH1) and NADH dependent (GDH2), which are located in chloroplasts and mitochondria, respectively. The expression of GDH1 and GDH2 was down-regulated in HN and LN. GS1 located in cytoplasm was gradually up-regulated with culture time, but GS2 located in chloroplast maintained a certain expression level in the early stage of culture and then was down-regulated, indicating that the reaction was transferred to cytoplasm in the late stage. The resulting glutamine (Gln) was then reduced to Glu catalyzed by glutamate synthase (or glutamate oxglutarate amino transferase, GOGAT). Similarly, GOGAT includes two types which were located in the cytoplasm (GOGAT1) and chloroplast (GOGAT2). The results showed that the gene of GOGAT1 was slightly up-regulated, and the gene of GOGAT2 was maintained. In heterotrophic culture, ammonia may be mainly metabolized by GO/GOGAT in *T. bernardii*.

## Conclusion

In this study, *Tetradesmus bernardii*, a newly isolated high-yielding oleaginous microalgal strain under photoautotrophic conditions, also exhibited high biomass and lipid accumulation in heterotrophic batch culture. The effect of NaNO_3_ on the increase of lipid content was much better than urea, and the highest lipid content of 45.0% of dry weight was achieved. Lipid accumulation could be further increased by extending the culture time. The biomass of *T. bernardii* in heterotrophic culture was closely related to nitrogen concentration and glucose concentration. A strong linear dependence was evident between the nitrogen concentration and biomass at certain concentrations of nitrogen and glucose. The rate of glucose to biomass exceeded 55% when the glucose concentration was less than 80 g/L in batch culture, which indicated *T. bernardii* had a relatively high glucose tolerance and great potential to be applied in biomass production in heterotrophic culture. Time-resolved transcriptome was conducted to elucidate C and N metabolism in heterotrophic culture of *T. bernardii.* As nitrogen was consumed in the medium, the nitrogen metabolism-related genes were significantly up-regulated to speed up the N metabolic cycle. The synthesis of starch was active which was the main storage C compound in heterotrophic culture, and the precursor of starch synthesis largely came from the absorption of the organic carbon source (glucose). Regarding lipid metabolism, the related genes of fatty acid synthesis at a low nitrogen concentration increased gradually with the extension of the cultivation time, while fatty acid degradation was also active, which may have resulted in a slow rate of lipid accumulation under heterotrophic metabolism.

## Materials and methods

### Algae strain and culture medium

*Tetradesmus bernardii* was identified and presented in Gao et al. [19]. The stock culture was maintained in a 250-mL flask containing 100-mL culture medium. The medium used in this heterotrophic experiment was modified Endo medium which contained glucose (10.0 g/L), (NH_2_)_2_CO (2.0 g/L), KH_2_PO_4_ (1.0 g/L), MgSO_4_·7H_2_O (1.0 g/L), CaCl_2_·2H_2_O (0.03 g/L), Sodium citrate (0.2 g/L), FeCl_3_·6H_2_O (3.15 mg/L), Na_2_EDTA·2H_2_O (4.36 mg/L), H_3_BO_3_ (2.86 mg/L), MnCl_2_·4H_2_O (1.81 mg/L), ZnSO_4_·7H_2_O (0.22 mg/L), Na_2_MoO_4_ (0.021 mg/L), and CuSO_4_·2H_2_O (0.08 mg/L). All medium components were heat sterilized (121 °C, 20 min).

### Cultivation conditions

In the heterotrophic culture of *T. bernardii*, NaNO_3_ and urea were used as nitrogen sources with nitrogen concentrations of 18 mM, and organic carbon source, glucose, was added into the medium at different concentrations (10, 20, 30, 40, 50, and 60 g/L). The cells were cultured in a 250-mL flask with working volume of 150 mL. The initial density of culture was 0.5 ± 0.05 g/L. Algae were kept in the dark at a constant culture temperature of 30℃ at 180 rpm. Algal culture was collected at day 12 and day 18 to prepare freeze-dried algal powder for subsequent lipid determination. All conditions were performed in triplicate.

With the glucose concentration of 60 g/L and NaNO_3_ used as the nitrogen source, the culture with different nitrogen concentrations of 4.5 mM, 9 mM, 12 mM, 15 mM, 18 mM, 27 mM, 36 mM, 45 mM, 54 mM, and 63 mM was conducted.

With the glucose concentration of 20 g/L and urea used as the nitrogen source, the culture with different nitrogen concentrations of 3 mM, 6 mM, 9 mM, 12 mM, 18 mM, 24 mM, and 30 mM was conducted. With the glucose concentration of 40 g/L and urea used as the nitrogen source, the culture with different nitrogen concentrations of 4.5 mM, 9 mM, 12 mM, 15 mM, 18 mM, 24 mM, 30 mM, 36 mM, 42 mM, 48 mM, 54 mM, and 60 mM was conducted. With the glucose concentration of 60 g/L and urea used as the nitrogen source, the culture with different nitrogen concentrations of 4.5 mM, 9 mM, 12 mM, 15 mM, 18 mM, 22.5 mM, 27 mM, 31.5 mM, 36 mM, 40.5 mM, 45 mM, 49.5 mM, 54 mM, 58.5 mM, and 63 mM was conducted. With the glucose concentration of 80 g/L and urea used as the nitrogen source, the culture with different nitrogen concentrations of 4.5 mM, 9 mM, 18 mM, 24 mM, 36 mM, 48 mM, 60 mM, 72 mM, 84 mM, 96 mM, 108 mM, and 120 mM was conducted. With the glucose concentration of 100 g/L and urea used as the nitrogen source, the culture with different nitrogen concentrations of 22.5 mM, 30 mM, 45 mM, 60 mM, 75 mM, 90 mM, 105 mM, and 120 mM was conducted. With the glucose concentration of 120 g/L and urea used as the nitrogen source, the culture with different nitrogen concentrations of 36 mM, 54 mM, 72 mM, 90 mM, and 108 mM was conducted.

With the glucose concentration of 60 g/L, nitrogen sources were NaNO_3_ and urea, with nitrogen concentrations of 15 mM and 45 mM. The growth and the absorption rate of glucose and nitrogen were measured by sampling every day for 9 days.

### Analysis method

#### Biomass measurement

10 mL of algal solution was filtrated through pre-weighed glass fiber filter membrane with 0.45 μm of pore size (dry weight, W1). The filter membranes containing algal cells were placed in an oven at 105 °C and dried to constant weight (dry weight, W2). The dry weight of the algae was calculated by the difference between W2 and W1, divided by 10 mL.

#### Lipid extraction

Total lipid was extracted by the method of Khozin-Goldberg et al. [37] with some modifications. 50–80 mg of freeze-dried algal powder was firstly extracted with 2 mL of dimethyl sulfoxide-methanol mixture (V:V = 1:9) in a water bath of 50 °C for 1.5 h, and then it was re-extracted with 4 mL of diethyl ether-hexane mixed solution (V:V = 1:1) in an ice bath for 1.5 h. The supernatant of extracting solution was collected into the same glass vial and the extraction process was repeated. Gravimetric method was used to determine lipid content and the detail of method was described in Gao et al. [19].

#### Total carbohydrate determination

10 mg of defatted algal residue was hydrolyzed with 5 mL of 0.5 M sulfuric acid at a constant temperature of 100 °C for 4 h. The total carbohydrate concentration was assessed quantitatively by the phenol–sulfuric acid method using glucose as reference [38]. The levels of residual glucose in the medium were also determined by the phenol–sulfuric acid method.

#### Fatty acid analysis

25 mg of algal powder was added into 2 mL of methanol containing 2% H_2_SO_4_ (V/V) in a 10-mL centrifuge tube, and 0.25 mg of heptadecanoic acid (Sigma Chemical Co., USA) was added, used as an internal standard. Then the centrifuge tube was filled with argon gas. The mixture was heated and stirred at 80 °C for 1.5 h. The fatty acid methyl esters (FAMEs) were analyzed with an Agilent Gas Chromatograph (Agilent 6890 N GC, Agilent Technologies, USA) and authentic standards. Detailed procedure has been described by Gao et al. [19].

#### Element analysis

About 300 μg of algal powder was folded into a tin cup (Elemental Microanalysis, Okehampton, UK), and cellular C and N were analyzed on a FLASH 2000 NC elemental analyzer (Brechbuhler Incorporated, Interscience B.V., Breda, The Netherlands).

#### RNA extraction, library construction, sequencing, assembly, and functional annotation

To learn the transcriptome changes of *T. bernardii* in heterotrophic culture under different C/N ratios, RNA-seq analysis was performed at glucose of 40 g/L with urea-nitrogen concentrations of 27 mM (HN) and 9 mM (LN), and then samples were taken at 0 h, 12 h, 2 d, 3 d, 5 d, 9 d, 12 d, and 15 d for each treatment. Total RNA of samples was extracted by method of RNAiso Plus (TaKaRa Biotech Co., Beijing, China). The detailed procedure of cDNA library construction has been described in Huang et al. [39]. RNA sequencing of each sample was conducted using an Illumina HiSeq 4000 by Beijing Genomics Institute (Shenzhen, China). The raw files were available from the NCBI SRA database under the accession number: PRJNA655830. The transcriptome was assembled using Trinity software. The assembled genes were annotated using the BLASTx with an E-value threshold of 1.0 E−5 against the databases as follows: NR (NCBI non-redundant protein sequences), COG (Clusters of Orthologous Groups of proteins), Swiss-Prot, KEGG (Kyoto Encyclopedia of Genes and Genomes), and GO (Gene Ontology).

#### Quantitative real-time polymerase chain reaction (RT-qPCR) analysis

To validate the expression levels of some of the genes, quantitative real-time polymerase chain reaction (RT-qPCR) analysis was performed using HN and LN samples. The qPCR primers (Additional file 3: Table S2) were designed by Primer Premier 6.0 software. The RT-qPCR was performed on CFX96 Touch (Bio-rad, Hercules, CA, USA) with PrimeScript™ RT reagent kit and TB Green™ Premix Ex Taq™ II (TaKaRa Biotech Co., Beijing, China) according to the manufacturer’s protocols. Samples were performed in triplicate. RT-PCR reactions were performed using a Step One Plus Real-Time PCR. The relative mRNA levels were normalized to the level of 18S rRNA gene in each sample and expressed as values of relative expression compared to that of the day 0 group. Relative levels of target mRNAs were determined using the 2^−ΔΔCt^ method and normalization [40]. The result of RT-qPCR analysis compared with transcriptome is presented in Additional file 1: Figure S10.

### Statistical analysis

Statistical analysis were performed using SPSS (version 22.0) statistical software. Significant differences (*P* < 0.05) between treatments were tested by one-way ANOVA (analysis of variance) (*P* < 0.05).

## Supplementary Information

**Additional file 1: Fig. S1.** Effect of different urea-nitrogen concentrations on the growth of *T. bernardii* at a glucose concentrations of 20 (a, b), 40 (c, d), and 60 g/L (e, f); (a, c, e) culture time of day 12; (b, d, f) culture time of day 18. **Fig. S2.** Effect of different urea-nitrogen concentrations on the growth of *T. bernardii* at a glucose concentrations of 80 (a, b), 100 (c, d), and 120 g/L (e, f); (a, c, e) culture time of day 12; (b, d, f) culture time of day 18. **Fig. S3.** Effect of different urea-nitrogen concentrations on the lipid content of *T. bernardii* at a glucose concentrations of 20 (a, b) and 40 g/L (c, d); (a, c) culture time of day 12; (b, d) culture time of day 18. **Fig. S4.** Effect of different urea-nitrogen concentrations on the lipid content of *T. bernardii* at a glucose concentrations of 60 (a, b) and 80 g/L (c, d); (a, c) culture time of day 12; (b, d) culture time of day 18. **Fig. S5.** Effect of different C/N molar ratios on the lipid productivity of *T. bernardii* at urea treatment; (a) culture time of day 12; (b) culture time of day 18. **Fig. S6.** Venn diagram of transcriptome annotation in *T. bernardii* between NR, NT, Swiss-Prot, KEGG, COG, and GO. **Fig. S7.** The annotation species distribution of transcriptome in *T. bernardii*. **Fig. S8.** The sum of different expression genes; (a) different expression genes of HN versus 0 h; (b) different expression genes of HN versus 0 h; (c) different expression genes of LN versus HN. **Fig. S9.** The top 20 up- and down-regulated metabolism pathways in *T. bernardii*; (a) top 20 of HN-up pathway enrichment; (b) top 20 of HN-down pathway enrichment; (c) top 20 of LN-up pathway enrichment; (d) top 20 of LN-down pathway enrichment. **Fig. S10.** Validation of mRNA-Seq-based transcript quantification using real-time quantitative PCR (qPCR).

**Additional file 2: Table S1.** Summary of sequencing data for each sample.

**Additional file 3: Table S2.** Primers for genes by Quantitative real-time polymerase chain reaction (RT-qPCR).

## Data Availability

The authors promise the availability of supporting data.

## Abbreviations

GLC: Glucose
HK: Hexokinase
HUP: H + /hexose cotransporter
G6P: Glucose-6-phosphate
G6PDH: Glucose-6-phosphate dehydrogenase
6-PGL: 6-Phosphogluconolactone
6-PG: 6-Phosphate gluconate
6PGDH: 6-Phosphogluconate dehydrogenase
Ru5P: Ribulose 5-phosphate
F6P: Fructose-6-phosphate
PFK: 6-Phosphofructokinase
FBP: Fructose-1,6-bisphosphatase
F-1,6-P: Fructose-1,6-bisphosphate
GAP: Glyceraldehyde-3-phosphate
G3P: Glycerate-3-phosphate
PEP: Phosphoenol pyruvate
PK: Pyruvate kinase
PYR: Pyruvate
PCK: Phosphoenolpyruvate carboxykinase
ACCoA: Acetyl-CoA (coenzyme A)
PC: Pyruvate carboxylase
AGP: ADP-glucose pyrophosphorylase
ADPG: ADP-glucose
SS: Starch synthase
BE: Branching enzyme
ACCase: Acetyl-coenzyme A carboxylase
BCCP: Biotin carboxyl carrier protein
BC: Biotin carboxylase
CT: Transcarboxylase
Lyso-PA: Lysophosphatidic acid
PA: Phosphatidic acid
PAP: Phosphatidic acid phosphatase
DAG: Diacylgycerol
GPAT: Glycerol-3-phosphate acyltransferase
LPAT: Lysophospholipid acyltransferase
DGAT: Diacylgycerol acyltransferase
TAG: Triacylglycerol
PLs: Phospholipids
PDAT: Phospholipid, diacylglcerol acyltransferase
Nrt: Nitrate transporter
NR: Nitrate reductase
NiR: Ferredoxin-nitrite reductase
GDH: Glutamate dehydrogenase
GS: Glutamine synthetase
Gln: Glutamine
Glu: Glutamate
GOGAT: Glutamate oxoglutarate amino-transferase
2-OG: 2-Oxoglutarate
