# Supplementary figures and images for: Biomass, lipid accumulation kinetics, and the transcriptome of heterotrophic oleaginous microalga Tetradesmus bernardii under different carbon and nitrogen sources
Source: Biotechnol Biofuels. 2021 Jan 6;14:4. doi: 10.1186/s13068-020-01868-9 (PMC7789750; doi:10.1186/s13068-020-01868-9)

Fig. S1

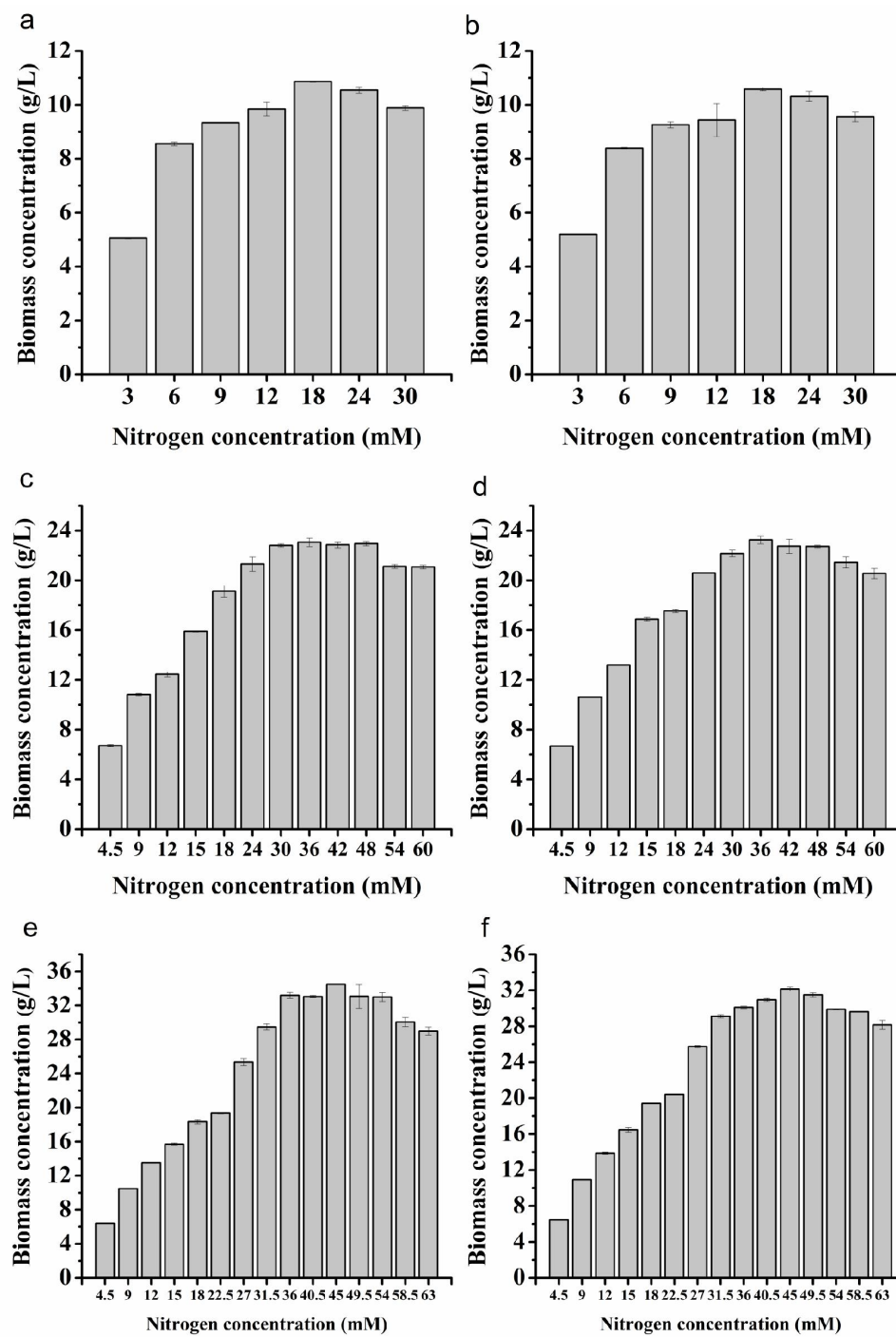

Fig. S2

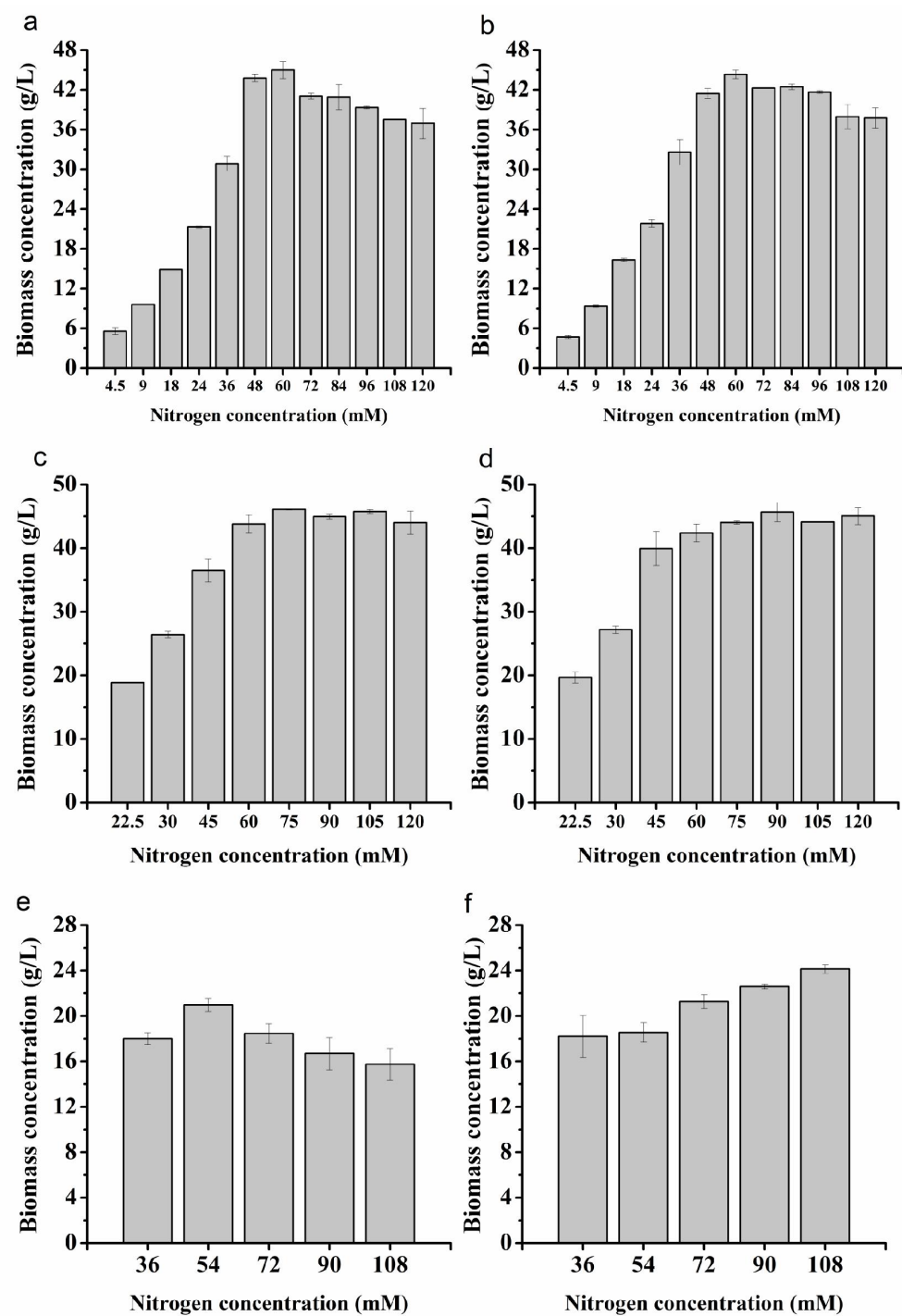

Fig. S3

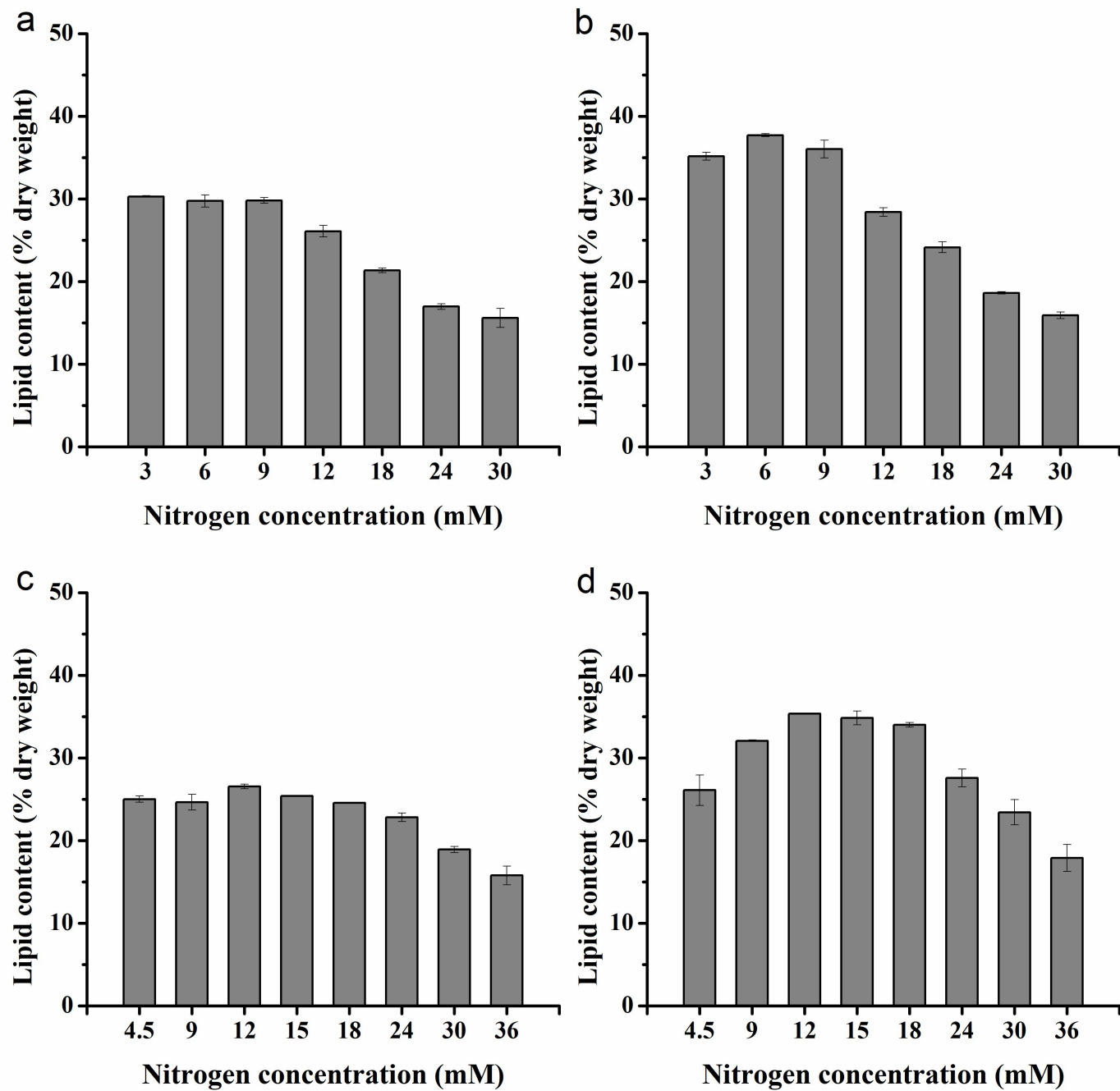

Fig. S4

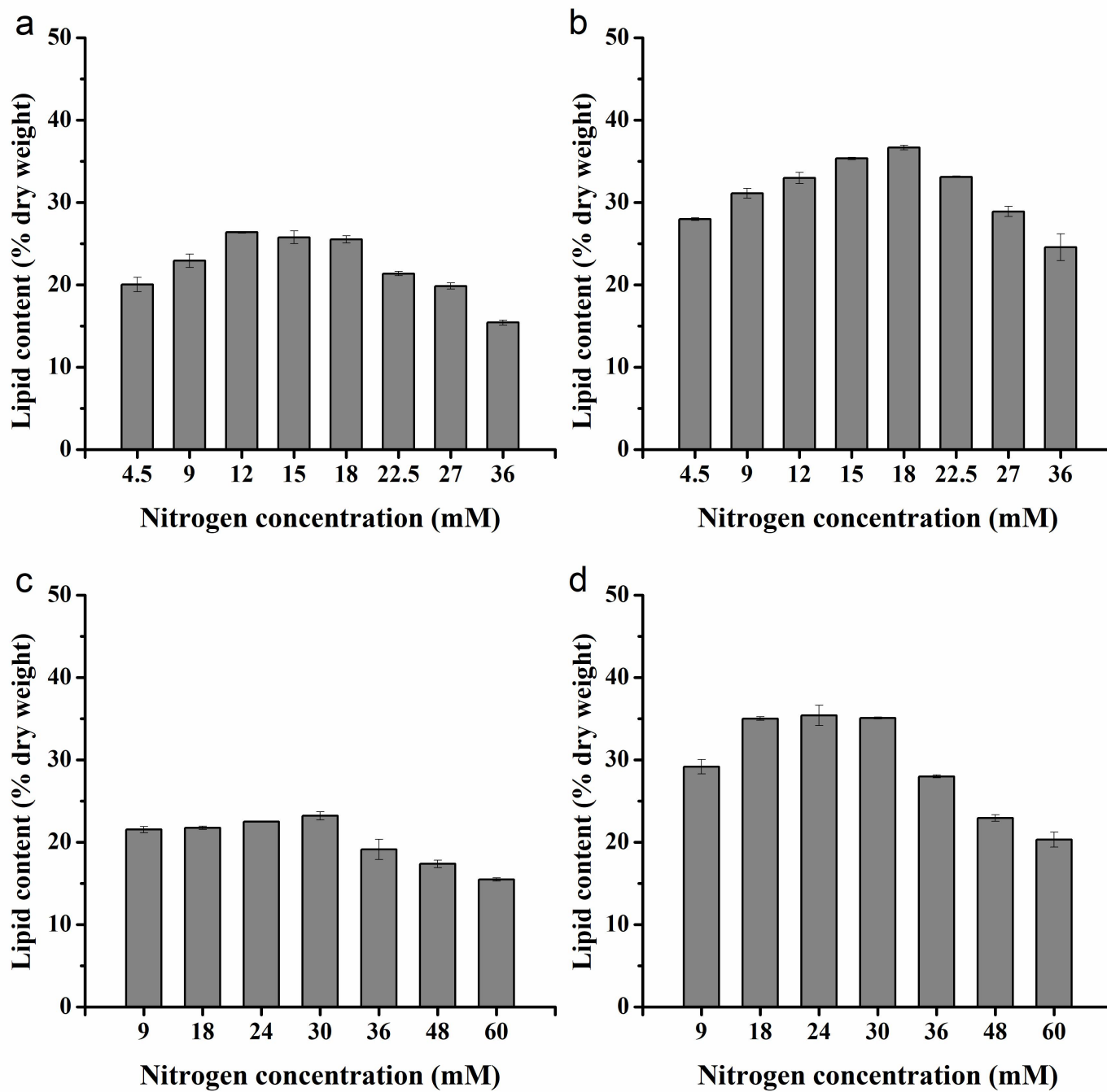

Fig. S5

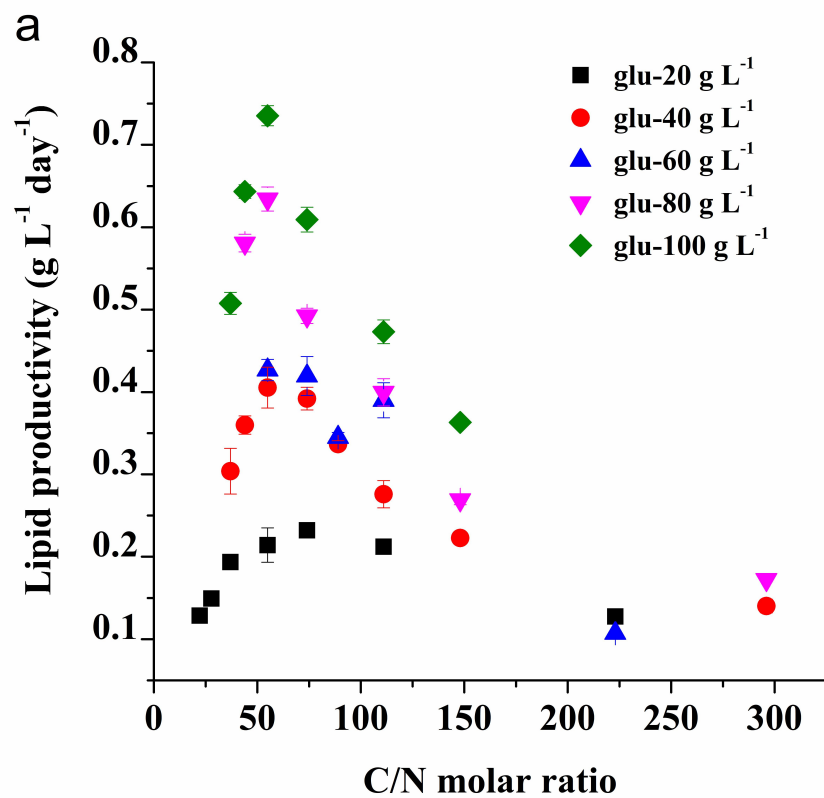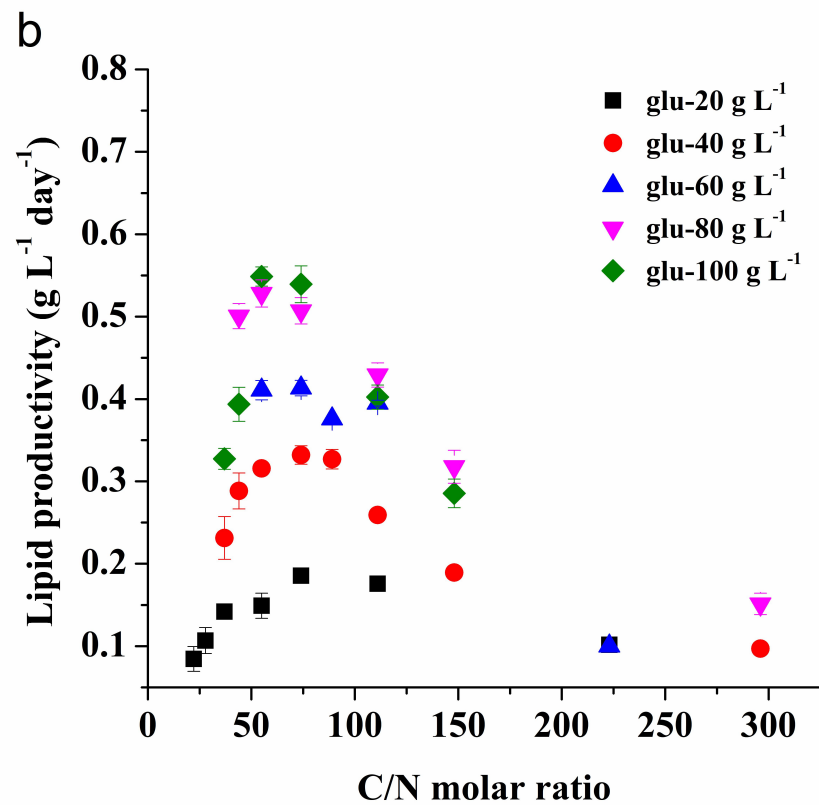

Fig. S6

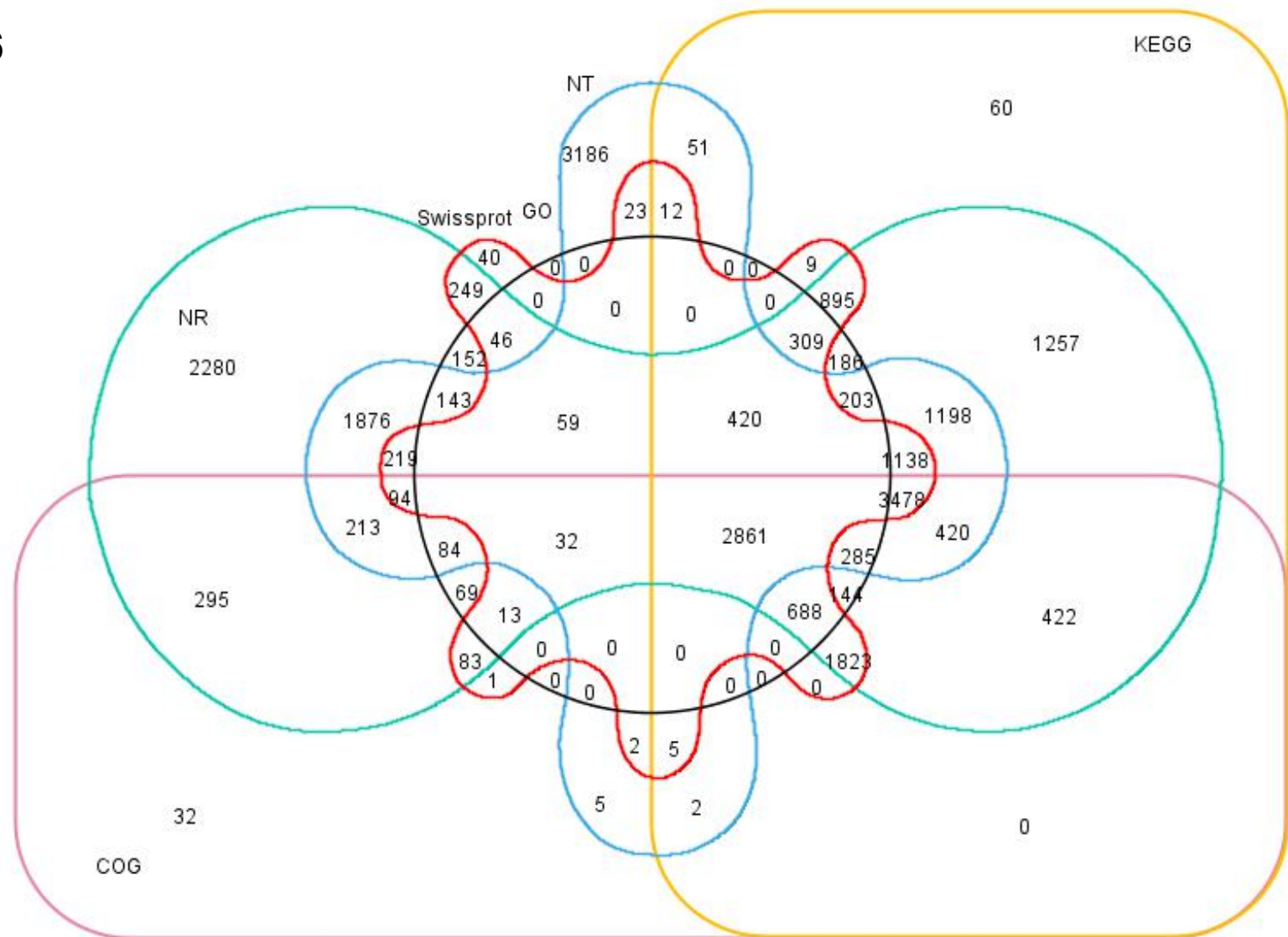

Fig. S7

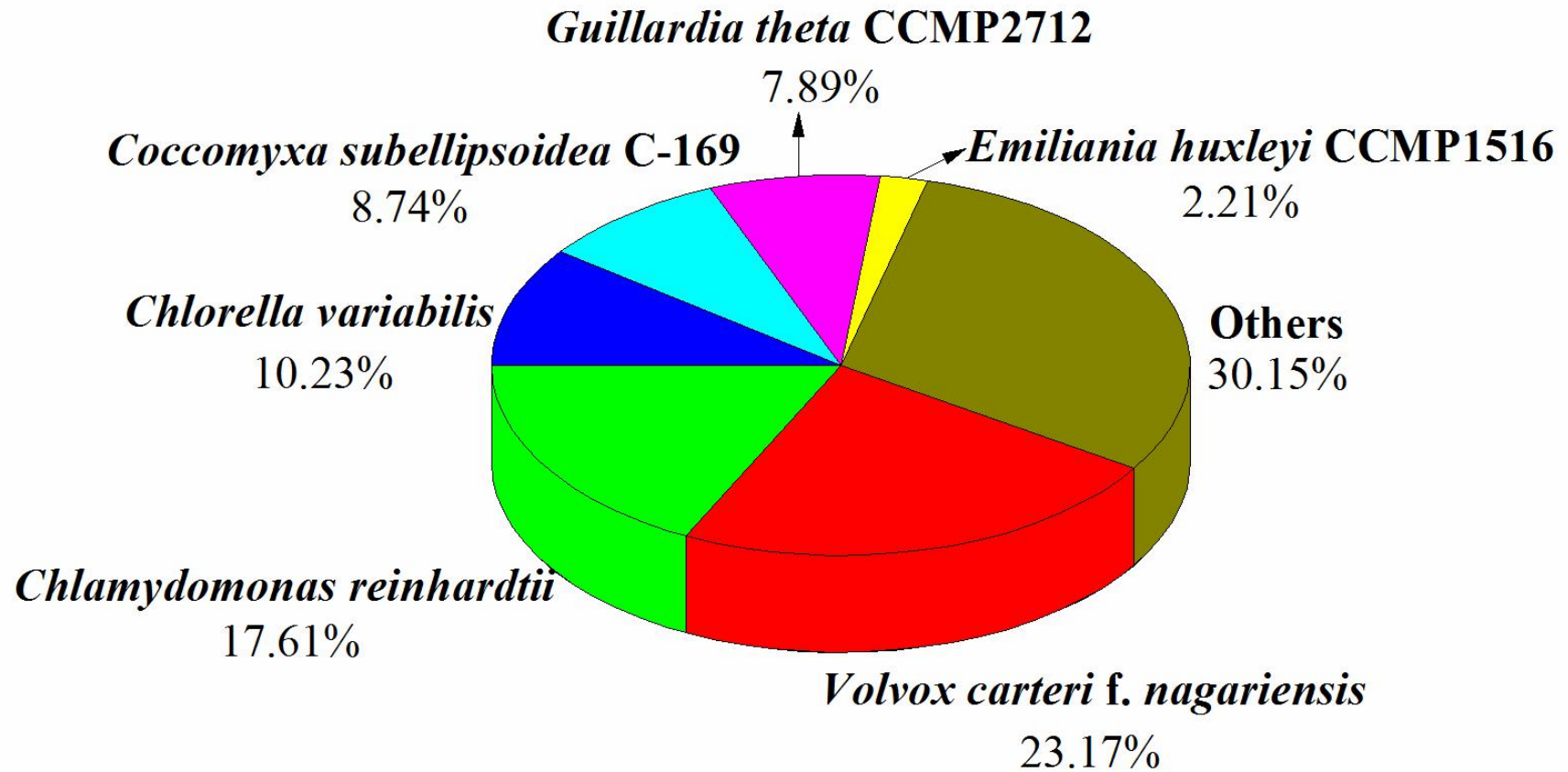

Fig. S8

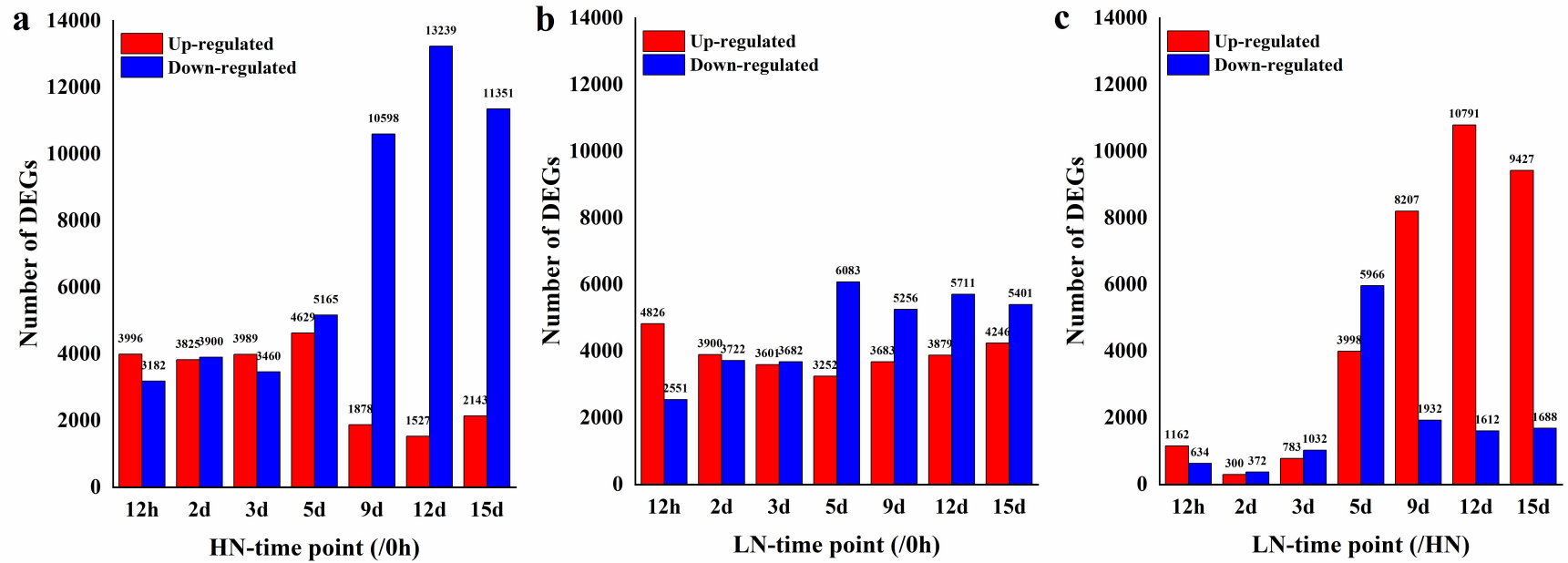

Fig. S9 a

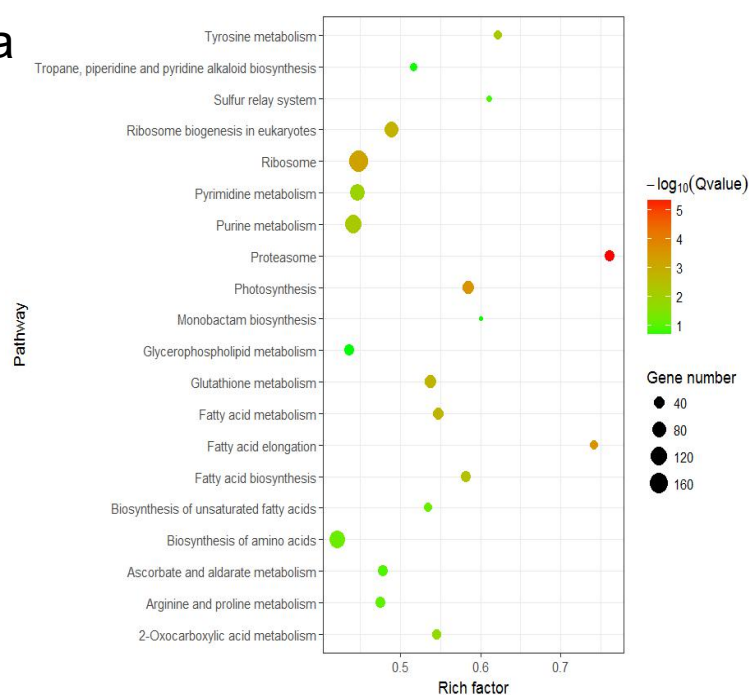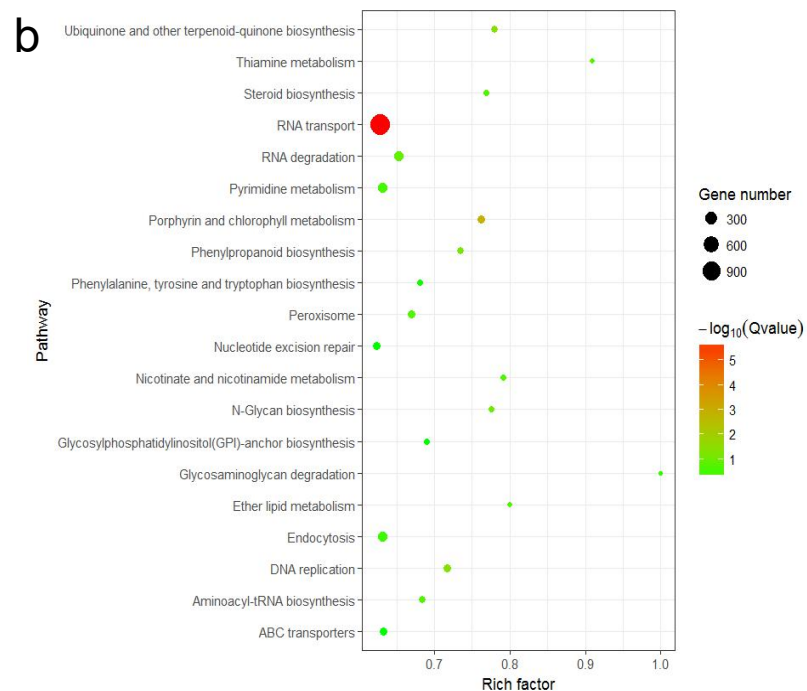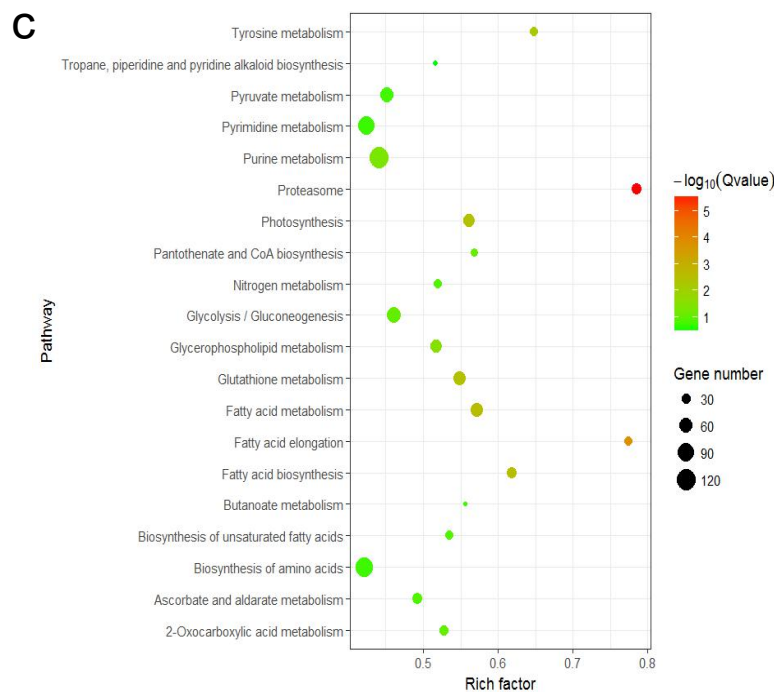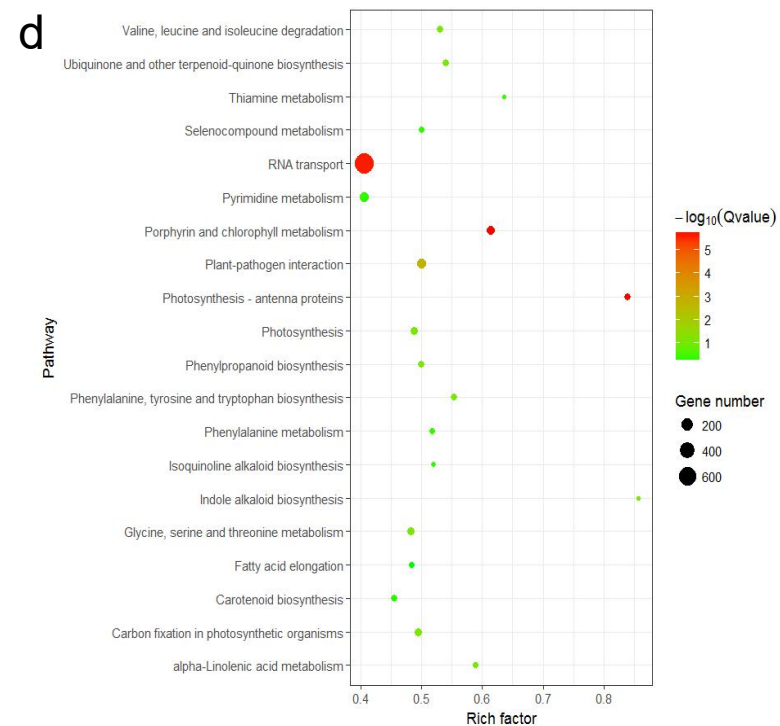

Fig. S9 a

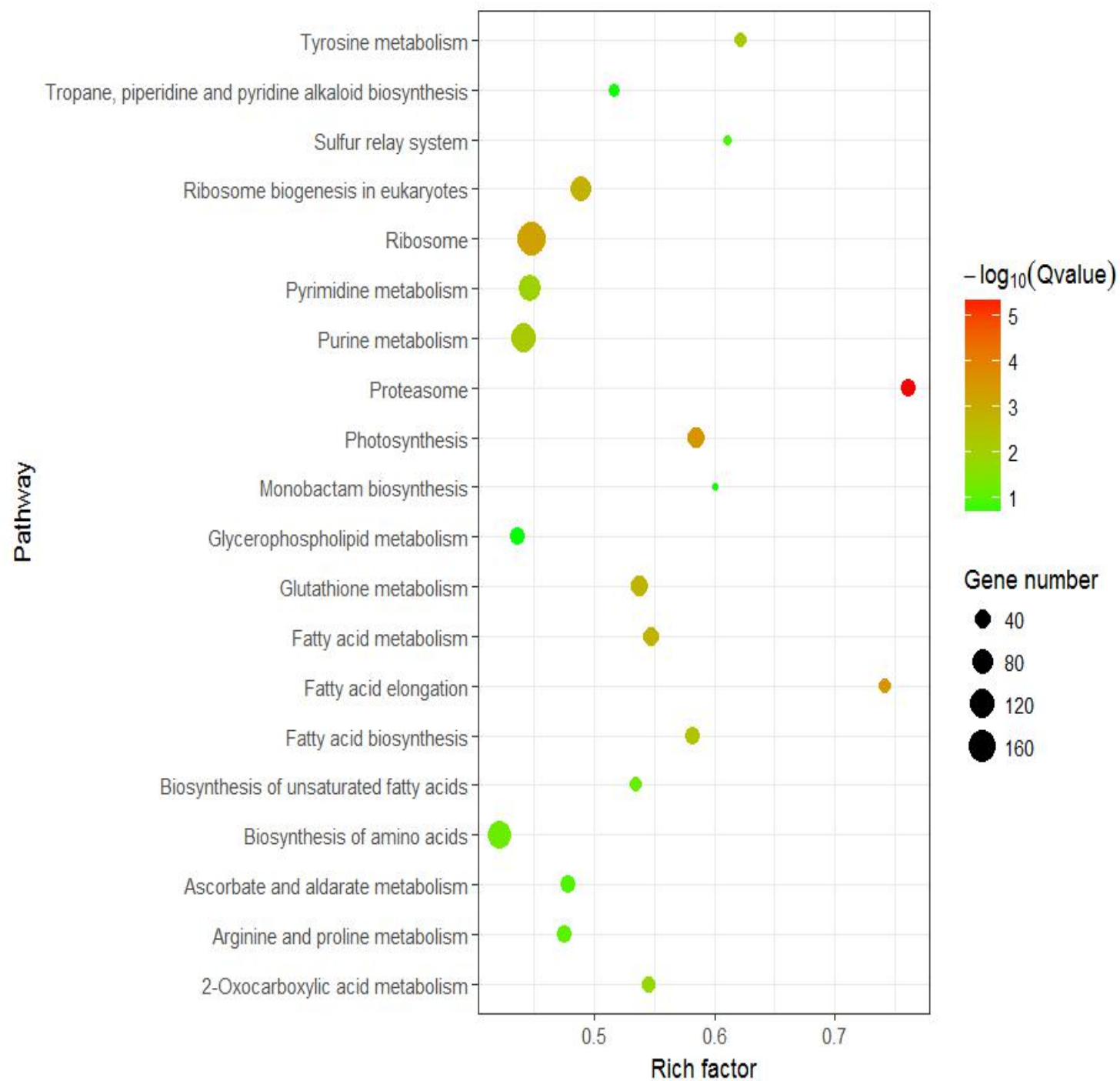

Fig. S9 b

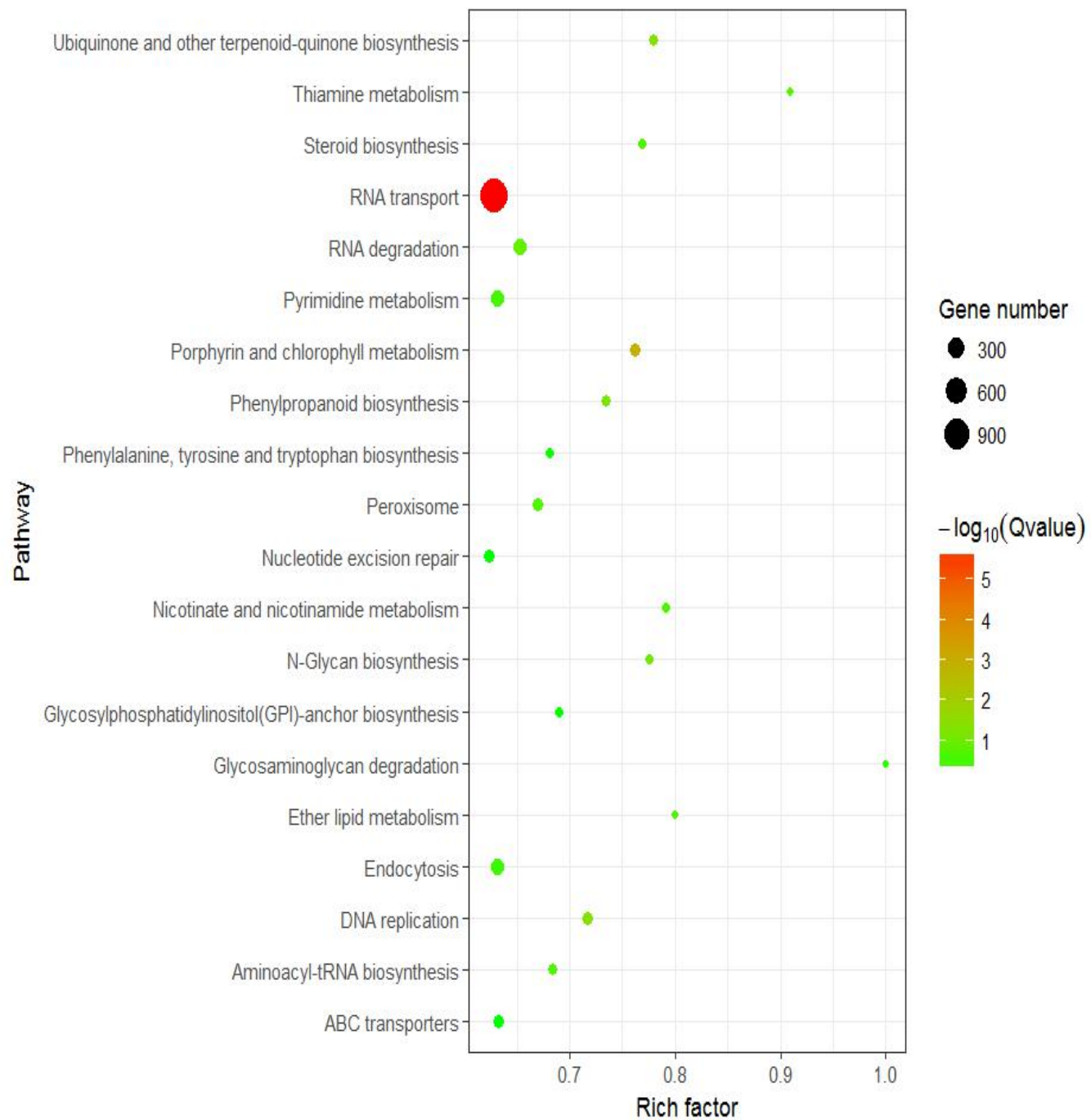

Fig. S9 c

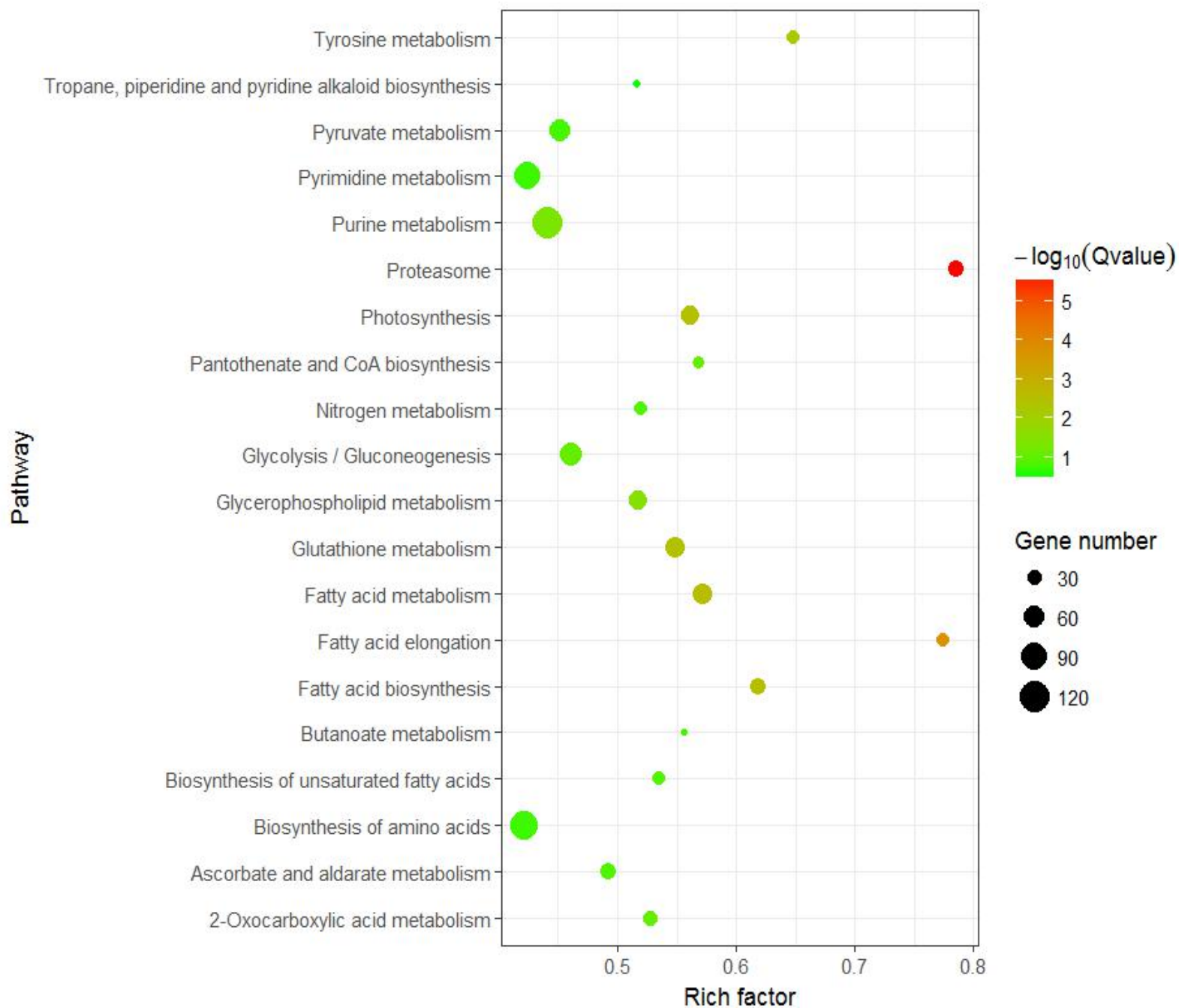

Fig. S9 d

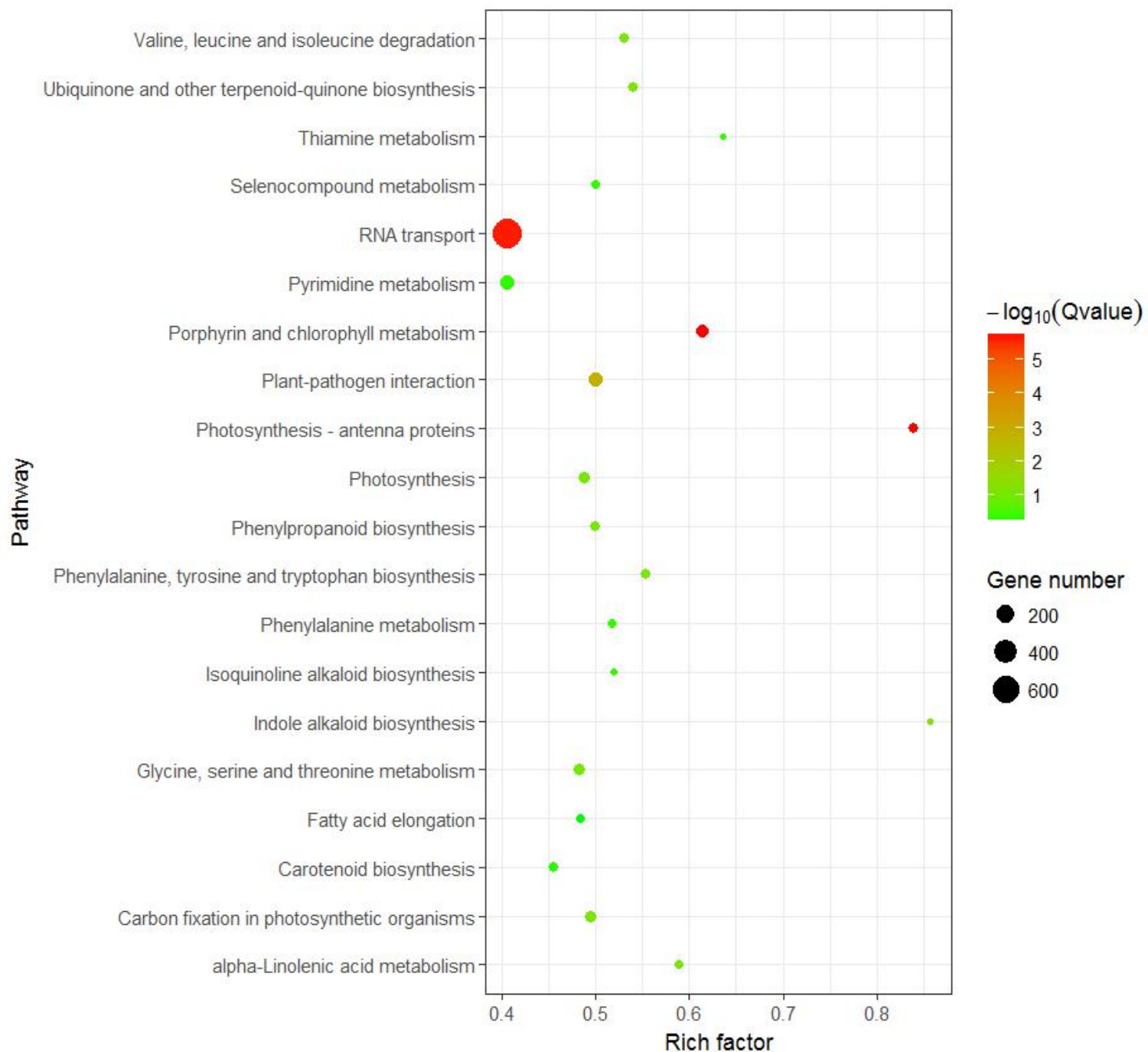

Fig. S10

a

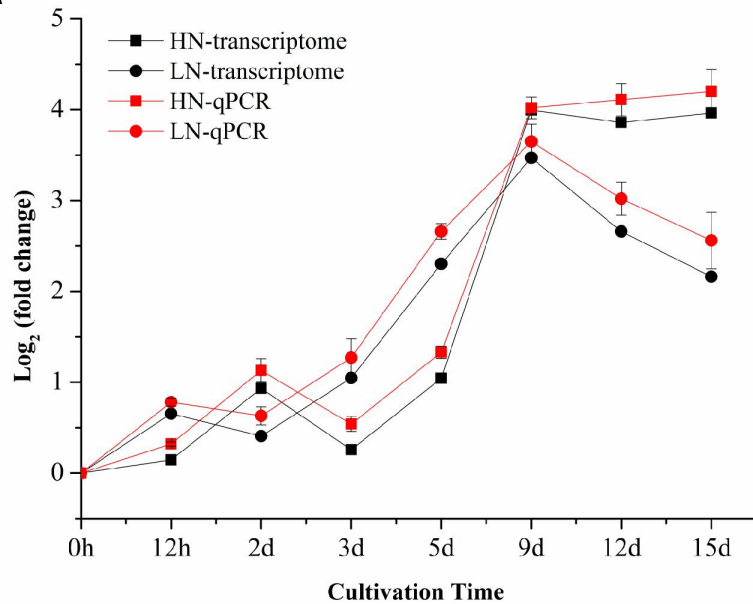

b

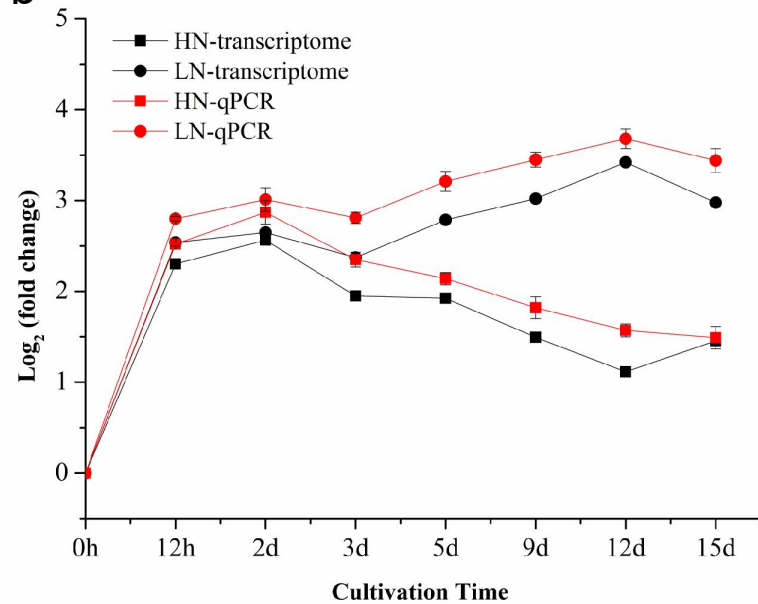

c

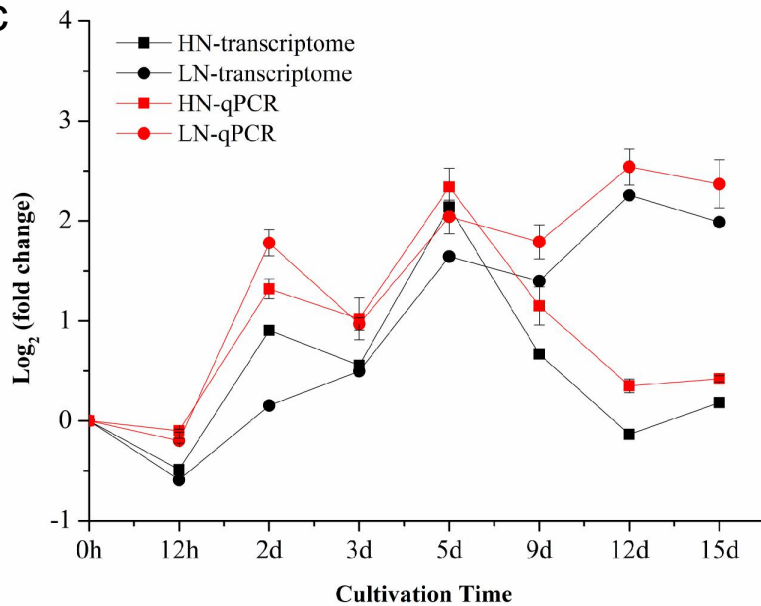

d

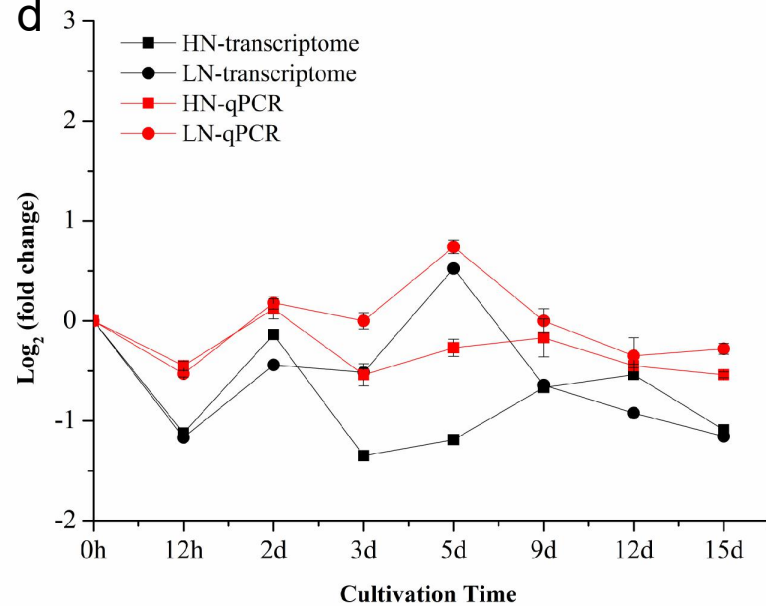

Supplement: Supplementary file 1 — Additional file 1: Fig. S1. Effect of different urea-nitrogen concentrations on the growth of T. bernardii at a glucose concentrations of 20 (a, b), 40 (c, d), and 60 g/L (e, f); (a, c, e) culture time of day 12; (b, d, f) culture time of day 18. Fig. S2. Effect of different urea-nitrogen concentrations on the growth of T. bernardii at a glucose concentrations of 80 (a, b), 100 (c, d), and 120 g/L (e, f); (a, c, e) culture time of day 12; (b, d, f) culture time of day 18. Fig. S3. Effect of different urea-nitrogen concentrations on the lipid content of T. bernardii at a glucose concentrations of 20 (a, b) and 40 g/L (c, d); (a, c) culture time of day 12; (b, d) culture time of day 18. Fig. S4. Effect of different urea-nitrogen concentrations on the lipid content of T. bernardii at a glucose concentrations of 60 (a, b) and 80 g/L (c, d); (a, c) culture time of day 12; (b, d) culture time of day 18. Fig. S5. Effect of different C/N molar ratios on the lipid productivity of T. bernardii at urea treatment; (a) culture time of day 12; (b) culture time of day 18. Fig. S6. Venn diagram of transcriptome annotation in T. bernardii between NR, NT, Swiss-Prot, KEGG, COG, and GO. Fig. S7. The annotation species distribution of transcriptome in T. bernardii. Fig. S8. The sum of different expression genes; (a) different expression genes of HN versus 0 h; (b) different expression genes of HN versus 0 h; (c) different expression genes of LN versus HN. Fig. S9. The top 20 up- and down-regulated metabolism pathways in T. bernardii; (a) top 20 of HN-up pathway enrichment; (b) top 20 of HN-down pathway enrichment; (c) top 20 of LN-up pathway enrichment; (d) top 20 of LN-down pathway enrichment. Fig. S10. Validation of mRNA-Seq-based transcript quantification using real-time quantitative PCR (qPCR). [file 13068_2020_1868_MOESM1_ESM.pdf]
