# Supplementary material for: Biomass, lipid accumulation kinetics, and the transcriptome of heterotrophic oleaginous microalga Tetradesmus bernardii under different carbon and nitrogen sources
Source: Biotechnol Biofuels. 2021 Jan 6;14:4. doi: 10.1186/s13068-020-01868-9 (PMC7789750; doi:10.1186/s13068-020-01868-9)
Supplement: Supplementary file 2 — Additional file 2: Table S1. Summary of sequencing data for each sample. [file 13068_2020_1868_MOESM2_ESM.doc]

| Sample | RNA-Seq Strategy | Clean Data Size (Gbp) | Raw Reads(Mb) | Clean Reads(Mb) | Clean Data Rate (%) | Clean Read Q20(%) | Total Mapped Reads (%) | Unique Match(%) |
| --- | --- | --- | --- | --- | --- | --- | --- | --- |
| Reference transcriptome | PE150 | 11.01 | 75.1 | 73.42 | 97.75 | 95.70 | NA | NA |
| 0h | SE50 | 1.12 | 23.02 | 23.01 | 99.96 | 96.60 | 94.61 | 80.42 |
| HN_12h | SE50 | 1.12 | 23.02 | 23.01 | 99.96 | 97.10 | 94.54 | 82.74 |
| HN_2d | SE50 | 1.12 | 23.02 | 23.01 | 99.96 | 96.70 | 94.25 | 81.39 |
| HN_3d | SE50 | 1.12 | 23.02 | 23.01 | 99.96 | 96.90 | 94.48 | 81.18 |
| HN_5d | SE50 | 1.12 | 23.02 | 23.01 | 99.98 | 97.40 | 92.96 | 79.58 |
| HN_9d | SE50 | 1.12 | 23.02 | 23.01 | 99.96 | 97.10 | 94.74 | 80.60 |
| HN_12d | SE50 | 1.12 | 23.02 | 23.01 | 99.95 | 97.90 | 95.02 | 80.15 |
| HN_15d | SE50 | 1.12 | 23.02 | 23.01 | 99.95 | 97.30 | 94.33 | 80.67 |
| LN_12h | SE50 | 1.12 | 23.02 | 23.01 | 99.98 | 96.50 | 94.12 | 82.79 |
| LN_2d | SE50 | 1.12 | 23.02 | 23.01 | 99.98 | 97.20 | 94.39 | 81.47 |
| LN_3d | SE50 | 1.12 | 23.02 | 22.97 | 99.8 | 96.20 | 94.16 | 81.00 |
| LN_5d | SE50 | 1.12 | 23.02 | 23.01 | 99.97 | 97.20 | 94.93 | 81.50 |
| LN_9d | SE50 | 1.12 | 23.02 | 23.00 | 99.92 | 97.00 | 94.63 | 81.48 |
| LN_12d | SE50 | 1.12 | 23.02 | 23.01 | 99.95 | 97.30 | 94.42 | 80.43 |
| LN_15d | SE50 | 1.12 | 23.02 | 23.01 | 99.96 | 96.60 | 94.27 | 80.48 |

Table S1 Summary of sequencing data for each sample
