# Supplementary material for: Biomass, lipid accumulation kinetics, and the transcriptome of heterotrophic oleaginous microalga Tetradesmus bernardii under different carbon and nitrogen sources
Source: Biotechnol Biofuels. 2021 Jan 6;14:4. doi: 10.1186/s13068-020-01868-9 (PMC7789750; doi:10.1186/s13068-020-01868-9)
Supplement: Supplementary file 3 — Additional file 3: Table S2. Primers for genes by Quantitative real-time polymerase chain reaction (RT-qPCR). [file 13068_2020_1868_MOESM3_ESM.doc]

Table S2 Primers for genes by Quantitative real-time polymerase chain reaction (RT-qPCR)

| Genes | Unigene ID | Primer | Sequence (5'–3′) | Product size (bp) |
| --- | --- | --- | --- | --- |
| 18S rRNA |  | 18S-F | CGGTCCGCCTATGGTGAGTA | 198 |
|  |  | 18S-R | CTCCGGTCCTACAGACCAACA |  |
| starch synthase | Unigene21074 | SS-F | GGCAATGTTGTGGATGCAGT | 128 |
|  |  | SS-R | GAGAGGACTGCGTGTTCGTG |  |
| pyruvate kinase | Unigene12413 | PK-F | TGCTCACACAACGAGGAGAG | 121 |
|  |  | PK-R | TTGCCATCTGGACCGCTATG |  |
| glucose 6-phosphate dehydrogenase | Unigene3815 | G6PDH-F | GCACTTGGCTGAGGATGAGA | 120 |
|  |  | G6PDH-R | TACTGCCTGCTCCAGAGTGG |  |
| diacylglycerol O-acyltransferase | CL825.Contig1 | DGAT2-F | ACGCTGTGTCAGGCAATCTG | 131 |
|  |  | DGAT2-R | TTGAAGTTGCCAGGTGTTGC |  |
